# Diagnostic accuracy of lung ultrasound score for bronchopulmonary dysplasia in preterm neonates: a systematic review and meta-analysis

**DOI:** 10.3389/fped.2025.1694150

**Published:** 2025-12-17

**Authors:** Chundi Han, Xiaohua Wang, Minli Pan, Xueyin Huang

**Affiliations:** 1 Department of Pediatrics, The First People’s Hospital of Xiaoshan District, Xiaoshan Affiliated Hospital of Wenzhou Medical University, Hangzhou, Zhejiang, China; 2 Department of Pediatrics, Ruian People’s Hospital, The Third Affiliated Hospital of Wenzhou Medical University, Wenzhou, Zhejiang, China

**Keywords:** bronchopulmonary dysplasia, diagnostic accuracy, lung, meta- analysis, ultrasonography

## Abstract

**Background:**

Bronchopulmonary dysplasia (BPD) remains common among preterm infants, yet diagnostic criteria rely on post-hoc oxygen requirements and radiography. Semiquantitative lung ultrasound (LUS) scoring offers a radiation-free, bedside alternative. We sought to determine the time-dependent diagnostic accuracy of LUS scores for BPD in preterm neonates.

**Methods:**

We performed a systematic review and meta-analysis of studies reporting LUS scores in preterm neonates (born at <37 gestational weeks) with BPD. We searched five databases through June 2025. We stratified data into four postnatal age timepoints (days 1–3, 7, 14, and 21) and pooled them using bivariate random-effects models to estimate sensitivity, specificity, likelihood ratios, diagnostic odds ratios, and area under the hierarchical summary receiver-operating characteristic (HSROC) curve.

**Results:**

We included data from 22 studies (*n* = 2,038 infants) in our analyses. Within days 1–3 (10 studies; *n* = 1,326), LUS scores yielded sensitivity of 0.75 (95% CI, 0.62–0.85), specificity of 0.74 (0.63–0.82), and AUROC of 0.81. At day 7 (15 studies; *n* = 1,790), sensitivity and specificity results improved to 0.78 (0.71–0.84) and 0.83 (0.78–0.87), respectively; and AUROC to 0.88. Day 14 performance was similar to that at day 7 (sensibility, 0.78; specificity, 0.84; AUROC, 0.87). By day 21 (4 studies; *n* = 619), the performance accuracy peaked [sensibility, 0.85 [0.73–0.92]; specificity, 0.86 [0.71–0.94]; and AUROC, 0.92]. Overall, positive likelihood ratios rose from 2.8 to 6.3 and negative ratios fell from 0.33 to 0.18. The heterogeneity decreased at the later intervals.

**Conclusion:**

The diagnostic accuracy of LUS scoring for BPD improved from the first through the third weeks of life, approaching optimal levels by day 21. These findings support the integration of serial LUS assessments into neonatal care plans to enable earlier, non-invasive BPD diagnosis.

**Systematic Review Registration:**

https://www.crd.york.ac.uk/PROSPERO/view/CRD420251085780, PROSPERO CRD420251085780.

## Introduction

Bronchopulmonary dysplasia (BPD) is one of the most important chronic lung diseases among survivors of preterm birth and remains a major cause of prolonged hospitalization, rehospitalization, and long-term respiratory and neurodevelopmental morbidity despite advances in perinatal care (1–4). Contemporary definitions, including the updated 2019 Neonatal Research Network criteria, primarily rely on standardized supplemental oxygen and respiratory support requirements at predetermined postnatal or postmenstrual ages and do not routinely require radiographic confirmation (3, 5). Although these operational definitions facilitate risk stratification and benchmarking across centres, they diagnose BPD relatively late in the clinical course and may not fully capture the underlying heterogeneity of lung injury.

Early and accurate identification of infants at high risk of BPD is essential to guide respiratory management, consider timely pharmacological interventions, and potentially mitigate long-term complications (6). Conventional imaging with chest radiography, and less frequently computed tomography, provides structural information but is limited by ionizing radiation, the need for transport out of the neonatal intensive care unit, and only moderate inter-observer reliability, particularly in early disease stages (7–9). In parallel, there is increasing interest in bedside tools and biomarkers that can predict BPD before formal diagnostic criteria are met, thereby enabling earlier and more individualized care strategies (4, 6, 10).

Lung ultrasound (LUS) has emerged as a promising radiation-free, point-of-care imaging modality in neonatal intensive care units (10–12). Semiquantitative LUS scores, derived from standardized scanning of predefined thoracic regions and grading of aeration loss, pleural irregularities, B-lines, and subpleural consolidations, offer an objective measure of pulmonary involvement (13, 14). The advantages of using LUS for predicting BPD include its capability for early detection well before 36 weeks’ postmenstrual age and its radiation-free nature, making it suitable for repeated bedside assessments in very preterm infants (10, 15, 16). Individual cohort studies have reported encouraging sensitivities and specificities for LUS-based prediction of BPD, but they differ in gestational age distributions, ultrasound protocols, timing of examinations, and reference standards, leading to heterogeneous and sometimes conflicting results (15, 16).

Two quantitative syntheses have previously evaluated LUS scores for early prediction of BPD, but important questions remain. Pezza et al. pooled seven relatively small cohorts and showed that different LUS scoring systems have broadly similar accuracy around 7–14 days of life, without systematically examining how test performance evolves later in the course or incorporating the many cohorts published more recently (17). Zhang et al. subsequently extended this work to 10 studies and focused mainly on the predictive value of a single LUS examination at day 7 (and, secondarily, day 14) for any and moderate–severe BPD (18). Neither meta-analysis was designed as a full diagnostic test accuracy review stratified across multiple postnatal windows, nor did they explore time-dependent changes in sensitivity, specificity, and likelihood ratios using hierarchical bivariate models. Our review was therefore conceived to fill this gap by updating the evidence base through June 2025, approximately doubling the pooled sample size, and quantifying the diagnostic performance of LUS scores at four distinct postnatal ages (days 1–3, 7, 14, and 21). This time-resolved analysis is intended to inform how and when LUS can be integrated into serial BPD surveillance and clinical decision-making in the neonatal intensive care unit.

## Methods

### Protocol and registration

We conducted this diagnostic test accuracy (DTA) systematic review and meta-analysis in accordance with the Preferred Reporting Items for Systematic Reviews and Meta-Analyses of Diagnostic Test Accuracy Studies (PRISMA-DTA) guidelines (19). A detailed protocol outlining objectives, eligibility criteria, search strategies, data extraction methods, and planned analyses was prospectively registered in PROSPERO (Registration No. CRD420251085780) prior to commencement of the literature screening.

### Eligibility criteria

#### Participants and target condition

We included studies enrolling preterm neonates (gestational age <37 weeks) assessed for BPD as defined according to consensus criteria (oxygen or respiratory support requirement). Studies of mixed age populations were eligible only if subgroup data for preterm infants were reported separately.

#### Index test

The index test is a semiquantitative LUS score, derived by scanning predefined thoracic regions and assigning scores based on the presence and extent of B-lines, consolidations, pleural irregularities, and “white lung” patterns. We accepted any LUS scoring system that mapped regional aeration loss to a numerical scale.

#### Reference standard

We used a BPD diagnosis standard comprising clinical and radiographic features and these established definitions: (1) NIH or any standard, guideline-based BPD definition based on oxygen and/or respiratory support requirements at specified time points; or (2) radiographic criteria on chest radiograph or computed tomography confirming BPD features. Studies using alternative but clearly defined BPD criteria were eligible if definitions were transparently reported.

#### Study design and timing

We included prospective or retrospective cohort, nested case–control, and randomized studies providing data to construct 2 × 2 diagnostic tables (true positives, false positives, false negatives, true negatives) for LUS scores vs. the reference standard. The studies included reported timing of LUS relative to the reference assessment. No language or publication-status restrictions were applied.

#### Exclusion criteria

We excluded case reports, case series with fewer than 10 infants, conference abstracts lacking full-texts, and studies in which LUS assessments were performed only after BPD diagnosis or without clear blinding to reference results.

### Information sources and search strategy

We developed a comprehensive search strategy with a medical librarian. We searched PubMed, Medline via Ovid, Scopus, Web of Science, and Cochrane Library databases from inception through June 2025. Search terms combined controlled vocabulary (e.g., MeSH: “Bronchopulmonary Dysplasia,” “Lung Ultrasonography”) and free-text keywords (“LUS score,” “preterm,” “diagnostic accuracy”). The full Boolean strategies for each database are listed in the Supplementary Appendix A1. We supplemented our electronic searches by hand-searching the reference lists of included studies and relevant reviews, and by querying clinical-trial registries for unpublished or ongoing studies.

### Study selection

We managed all retrieved records in EndNote and uploaded them into Covidence for de-duplication. Two reviewers independently screened titles and abstracts against the eligibility criteria. Full-text articles were obtained for records deemed potentially eligible by either reviewer. These were then assessed in duplicate before final inclusion. Discrepancies at both screening stages were resolved by discussion or, if necessary, adjudication by a third senior reviewer. We created a PRISMA flow diagram (Supplementary Figure S1) with study selection process details.

### Data collection process

Using a pilot-tested data-extraction form, two reviewers independently abstracted study-level data in duplicate. Extracted items included: study setting and design, inclusion/exclusion criteria, sample size, gestational age and birth-weight characteristics, LUS protocols (regions scanned, scoring rubric, examiner training and blinding), reference standard definitions, and timing, threshold(s) for test positivity, and 2 × 2 contingency data. We also recorded covariates potentially affecting the diagnostic performance of the technique (e.g., use of antenatal steroids, ventilation mode). Disagreements were reconciled by consensus discussion.

### Risk of bias and applicability assessment

We assessed the methodological quality of the studies included using the Quality Assessment of Diagnostic Accuracy Studies-2 (QUADAS-2) tool, structured across four domains: patient selection, index test, reference standard, and flow and timing (20). Two reviewers rated each domain for risk of bias (“low,” “high,” or “unclear”) and applicability concerns. We made judgments about study design, test conduct, blinding, and attrition on the basis of signalling questions. Discrepancies were resolved through discussion or by consulting a third reviewer.

### Diagnostic accuracy measures

For each study, we calculated sensitivity, specificity, positive and negative likelihood ratios (LR+ and LR–), and diagnostic odds ratio (DOR) at reported LUS-score thresholds. In cases in which multiple thresholds had been evaluated, we selected the primary threshold defined by the authors; and we explored secondary thresholds in subgroup analyses. We also captured values of area under the summary receiver-operating characteristic curve (AUCs) when provided.

### Data synthesis and statistical analysis

We synthesized diagnostic accuracy estimates using a bivariate random-effects model, which jointly models sensitivity and specificity while accounting for their correlation and between-study heterogeneity. We generated pooled summary estimates with 95% confidence intervals and constructed a hierarchical summary receiver-operating characteristic (HSROC) curve. Heterogeneity was quantified by variance parameters (*τ*^2^) and visual inspection of the HSROC plot. We performed the analyses of the defined timepoints (first three days of life, day 7, day 14, day 21, and day 28) as per the LUS timing. We used a bivariate box plot and I-squared statistic to check for heterogeneity. We assessed publication bias using Deeks’ funnel-plot asymmetry test, with *p* < 0.10 indicating potential bias. Subgroup analysis was performed based on gestational age. All statistical analyses were conducted in Stata 17 (StataCorp, College Station, TX) using the “midas” and “metandi” commands for DTA meta-analyses (21).

We plotted likelihood-ratio (LR) scattergrams to display the distribution of study-specific positive and negative LRs and to assess the test's clinical utility across different scenarios. In the LR scattergram, points in the left upper quadrant (LR^+^ ≥ 10 and LR^−^ ≤ 0.1) indicate tests that reliably either confirm or exclude the disease, demonstrating excellent rule-in and rule-out performance. Points in the right upper quadrant (LR^+^ ≥ 10 and LR^−^ > 0.1) denote strong rule-in ability but insufficient rule-out power, making the test useful for confirmation only. Conversely, points in the left lower quadrant (LR^+^ < 10 and LR^−^ ≤ 0.1) reflect good rule-out capacity but limited confirmation utility, favouring exclusion. Finally, points in the right lower quadrant (LR^+^ < 10 and LR^−^ > 0.1) reflect poor discrimination of the LUS scores, lacking both adequate rule-in and rule-out capabilities. We build Fagan nomograms using the pooled LR+ and LR– to translate a range of pre-test probabilities into post-test probabilities, illustrating the impact of LUS scores on diagnostic decision-making.

## Results

### Search results

Supplementary Figure S1 depicts the study selection process in accordance with PRISMA-DTA guidelines. We identified a total of 2,108 records through the database searches (PubMed, *n* = 723; Scopus, *n* = 492; MEDLINE via Ovid, *n* = 398; Cochrane Library, *n* = 121; and Web of Science, *n* = 374) and removed 512 duplicates, yielding 1,596 unique records for title and abstract screening. After full-text review of 115 reports, we excluded 93 for reasons that included the use of a different index test (*n* = 65), non-eligible participants (*n* = 26), or unavailability of analysable data (*n* = 2), leaving 22 studies for inclusion in the final analysis (15, 22–42).

### Characteristics of the included studies

Table 1 presents key characteristics of the 22 included studies, which collectively enrolled 2,038 preterm infants across North America, Europe, Asia, and the Middle East. Most studies were prospective cohorts (*n* = 20) and two were retrospective cohort studies. Sample sizes ranged from 27 to 298 neonates. Gestational ages spanned from 23 to 34 weeks, with birth weights from medians of 690 g to means exceeding 1,500 g. LUS scoring protocols varied from 6-zone to 12-zone examinations, employing semiquantitative scales (0–3 or 0–4 per region), and total scores between 18 and 36. The imaging timepoints ranged between 1 h after birth and 36 weeks PMA. BPD definitions were based on NIH/NICHD consensus criteria at 28 days after birth or 36 weeks PMA, oxygen dependency, or physiologic grading, ensuring consistent outcome ascertainment. The results of our risk-of-bias assessment classified more than half of the studies (13 studies) as having low risk of bias reflecting overall methodological robustness, five as having moderate risk, and four as having high risk (primarily due to retrospective design or incomplete blinding).

**Table 1:** Characteristics of included studies (*N* = 22).

Study (Year)	Region	Study design	Characteristics of participants	Gestational age range	Birth weight (Median, IQR)	Mean age at LUS (Hours)	BPD definition	LUS protocol used	Sample size	Risk of bias
Abdelmawla et al. (2019) (22)	Canada & Saudi Arabia	Retrospective	Preterm infants <30 weeks GA on respiratory support	25–29 weeks	Median: 780 (IQR, 530–1,045)	Median: 35 (5 weeks)	Need for positive pressure/supplemental O₂ at 36 weeks postmenstrual age (PMA)	LUS with 3 lung zones/side; score, 0–3 per zone; max score, 18	27	High
Abdelrazek et al. (2024) (15)	Egypt	Prospective cohort	Preterm neonates ≤34 weeks on oxygen therapy during first 7 days	27–34 weeks	1,347.66 ± 432.14 g	Day 7 and Day 14	Oxygen requirement at 28 days of life and at discharge	8-zone LUS; score 0–3 per zone; total possible score, 0–24	96	Low
Aliyev et al. (2024) (24)	Turkey	Retrospective cohort	Preterm infants <32 weeks GA admitted to NICU within first 3 days	30.4 (non-BPD) to 26.4 (severe BPD)	1,495 (non-BPD) to 736 g (severe BPD)	Within first 3 days	Oxygen requirement at 36 weeks PMA classified as mild/moderate/severe BPD	6-zone LUS (3 per lung); score, 0–3 per zone; total max score, 18	218	Moderate
Hoshino et al. (2022) (29)	Japan	Prospective cohort	Preterm infants <32 weeks GA on O₂ therapy at 28 days of life	29.4 ± 2.4 weeks	1,135 g (IQR, 898–1,403)	Day 28	NIH definition; moderate: O_2_ < 30%, severe: CPAP or ventilation at 36 weeks PMA	Whole chest (6 zones: 2× anterior, lateral, posterior); score, 0–3/zone; max score, 18	87	Low
Li et al. (2023) (31)	China	Prospective cohort	Preterm infants <32 weeks GA admitted to NICU from birth	28.7 ± 1.6 weeks	1,176.5 ± 224.6 g	Days 3, 7, 14, and 21	Oxygen or respiratory support at 36 weeks PMA (*N*IH criteria)	6-region LUS (3 per lung); score 0–3/region; total score, 0–18	150	Low
Liu et al. (2021) (32)	China	Prospective cohort	Very low birth weight infants (<32 weeks GA), admitted within 24 h	29.2 ± 1.8 weeks	1,195.0 ± 209.8 g	Days 1–15 (every 3 days after Day 3)	NIH 2001 & 2019 definitions (based on O_2_ or respiratory support at 36 weeks PMA)	6-, 10-, and 12-region LUS protocols; score, 0–3/region; total max, 36 (for 12-region)	130	Low
Loganathan et al. (2025) (33)	United Kingdom	Prospective multicentre cohort	Preterm infants ≤34 weeks on non-invasive respiratory support within 3 hours of birth	≤34 weeks	1,515 g (IQR, 1,185–1,950)	1.93 (IQR 1.4–2.4)	O_2_ or respiratory support at 36 weeks PMA (NIH 2001)	6- and 10-zone LUS; score, 0–3 per zone; max scores, 18 (6-zone), 30 (10-zone)	83	Low
Khandelwal et al. (2025) (30)	India	Prospective observational study	Preterm neonates ≤30 weeks GA, required ≥24 h respiratory support	24–30 weeks (mean GA BPD: 28.3 ± 1.51; non-BPD: 29.18 ± 0.99)	BPD: 923 g ± 229; Non-BPD: 1,181 g ± 231	First scan 24–72 h; then weekly	NHLBI 2001	6-zone LUS (Brat et al.)	112	Moderate
Raimondi et al. (2021) (39)	Italy	Prospective multicentre	Preterm infants with RDS, GA 25–33w	25–33 weeks	25–27w: 865 g ± 165; 28–30w: 1,140 g ± 284; 31–33w: 1,682 g ± 373	<72 h for baseline, 7d for BPD prediction	NR	Brat et al. modified, lateral + posterior zones	240	Moderate
Gao et al. (2020) (27)	China	Prospective observational	Preterm ≤28 weeks GA or ≤1,500 g birth weight	≤28 weeks	BPD: 1,079.7 ± 165.3 g	3, 7, 14, 28 days	NICHD 2001	12-zone scoring	81	Low
Alonso-Ojembarrena et al. (2021) (25)	Spain	Multicentre Prospective Observational	Preterm infants <32 weeks; no major anomalies; 298 included	29 (26–30) weeks	1,100 g (859–1,340 g)	Day 0, 3, 7, 14, 21	Physiologic definition	Anterolateral & posterior (compared)	298	Low
Alonso-Ojembarrena et al. (2019) (26)	Spain	Prospective cohort	Very low birth weight infants (≤1,500 g and/or ≤32 weeks GA)	23–32 weeks	BPD: 914 g (461–1,367), Non-BPD: 1,241 g (837–1,440)	1st, 3rd day, weekly until 36 weeks PMA	NIH definition; includes mild, moderate, and severe BPD based on O₂ at 36 wks PMA	LU at 6 zones (3 per lung); score, 0–3 each; total score, 0–18; assessed serially	59	Moderate
Loi et al. (2021) (34)	France & Italy	Prospective longitudinal cohort	Preterm infants (≤30 + 6 weeks GA)	Mean: 27.3 ± 1.9 weeks	954 ± 289 g	Days 0, 7, 14, 28, and 36 weeks PMA	NIH 2,001: O₂ or respiratory support at 36 weeks PMA	6-zone and 10-zone LUS; gestational age–adjusted scoring (LUS and eLUS); semiquantitative 0–3/zone	147	Low
Martini et al. (2023) (35)	Italy	Prospective bicentric cohort	Preterm infants ≤34 weeks GA with RDS admitted to NICU	Median: 30.7 (IQR: 28.4–32)	1,289 g (IQR, 979–1,642)	Days 1–3 (within 72 h)	Oxygen or respiratory support at 36 weeks PMA (NIH 2001 definition)	6-zone LUS; score, 0–3/zone; total score, 0–18; evaluated daily for 3 days	64	High
Mohamed et al. (2021) (36)	Canada	Prospective cohort (2-center)	Preterm infants <29 weeks GA	Mean: 25.8 ± 1.5 weeks	Mean: 923 ± 250 g	Days 3, 7, and 14	NIH 2001 (O₂ or respiratory support at 36 weeks PMA or discharge)	6-zone (3 per lung); LUS score, 0–18; score >10 predictive at all timepoints	152	Low
Oulego-Erroz et al. (2021) (37)	Spain	Prospective observational	Preterm infants <32 weeks GA admitted to NICU	<32 weeks	Non-sBPD: 1,235 (1,110–1,507), sBPD: 890 (655–1,130)	7 days	NIH 2001: moderate (O_2_ <30%) or severe (O_2_ ≥ 30% or PPV) at 36 weeks PMA	8-zone (4 per lung); score, 0–3 per zone; total score, 0–24	42	Low
Palacio et al. (2025) (38)	Spain	Prospective longitudinal	Preterm infants born <30 weeks GA admitted to NICU	23.0–29.6 weeks	No BPD: 993 [840–1,054], BPD: 690 [575–913]	DOL 0 (1–2 h), 3, 7, 28, and 36 wks PMA	Respiratory support mode at 36 wks PMA	6-zone, LUS 0–18; Brat et al. scoring; evaluated serially	42	Moderate
Aldecoa-Bilbao et al. (2021) (23)	Spain	Prospective observational	Very Preterm Infant ≤30.6 weeks GA	23–30.6 weeks	BPD: 853 ± 252 g Non-BPD: 1,163 ± 314 g	7 days (also admission, 28d)	NICHD 2001	6-zone protocol (anterior, lateral, posterior)	89	Low
Ghanem et al. (2024) (28)	Canada	Secondary analysis of prospective study	Preterm ≤28 weeks GA	24–28 weeks	BPD group: 829.6 g (SD 251.6) Non-BPD: 997.6 g (SD 215.8)	Day 3 (72 h), 7d, 14d	O2/support dependency at 36w PMA	6-zone; score, 0–18	132	High
Sun et al. (2022) (40)	China	Prospective diagnostic accuracy	Preterm infants <34 weeks GA, admitted <28 days of life	Mean: 28.8 ± 2.3 weeks	1,218 ± 392 g	36 weeks PMA	NICHD/NHLBI 2001: severity classified as mild, moderate, severe	mLUS: 8-zone (6 chest + 2 lung base zones); score, 0–3 per zone; total score, 0–24	128	High
Szymański et al. (2024) (41)	Poland	Prospective multicentre	Preterm infants <34 weeks GA with RDS requiring non-invasive support	Median: 32 (IQR: 30–33)	1,660 ± 495 g	Day 1 (0–6 h), Day 2, 3, 7	NIH 2001: O_2_ ≥ 28 days, graded at 36 wks PMA or Day 56	0–16 score system, anterior & posterior zones, blinded centralized rating	136	Low
Zong et al. (2024) (42)	China	Prospective observational	Preterm neonates ≤25 + 6 weeks GA admitted to NICU	≤25 weeks	698 g (BPD), 742 g (non-BPD)	Day 14	NICHD 2018 BPD and moderate–severe BPD	12-zone scan (6 zones per side); modified Brat scoring scale, 0–4; anterior, lateral, posterior	89	Low

GA, gestational age; NICU, neonatal intensive care unit; O₂, oxygen; CPAP, continuous positive airway pressure; PMA, postmenstrual age; RDS, respiratory distress syndrome; VPI, very preterm infant; BPD, bronchopulmonary dysplasia; sBPD, severe bronchopulmonary dysplasia; msBPD, moderate-to-severe bronchopulmonary dysplasia; LUS, lung ultrasound score; eLUS, extended lung ultrasound score; IQR, interquartile range; SD, standard deviation; NHLBI, National Heart, Lung, and Blood Institute; NIH, National Institutes of Health; mLUS, modified lung ultrasound score; DOL, days of life; NR, not reported.

### Diagnostic accuracy of LUS score for BPD during the first three days of life

**Figure 1:**
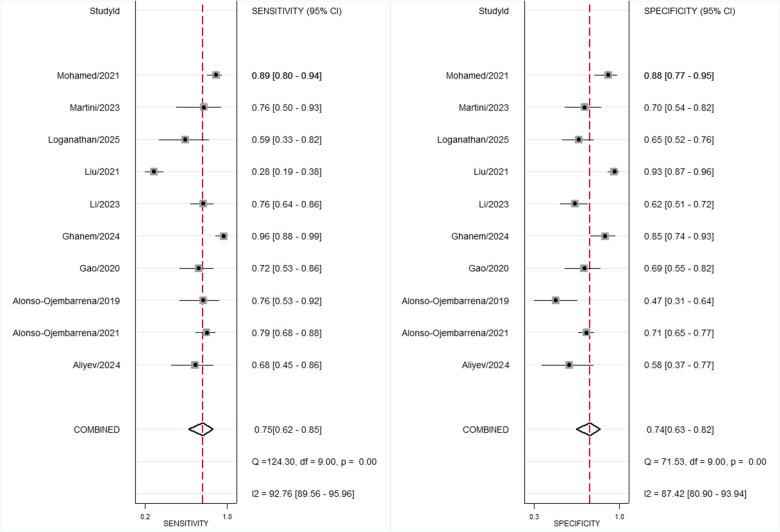
Forest plot for diagnostic accuracy of lung ultrasound scores for bronchopulmonary dysplasia at first three days of life.

**Figure 2:**
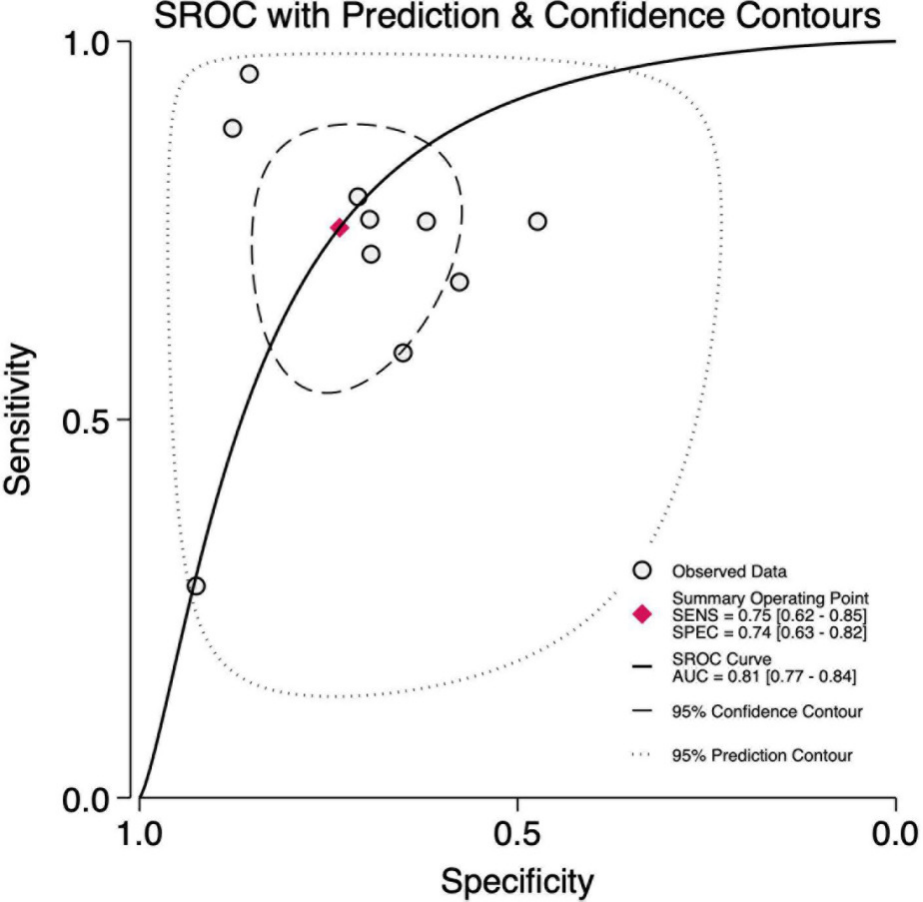
Summary receiver operator characteristics curve plot for diagnostic accuracy of lung ultrasound scores for bronchopulmonary dysplasia at first three days of life.

Across ten studies including 502 BPD-positive and 824 BPD-negative preterm infants in the first three days of life, the pre-test probability of disease was 38%. The bivariate meta-analysis (Figure 1) yielded a pooled sensitivity of 0.75 (95% CI, 0.62–0.85) and a pooled specificity of 0.74 (95% CI, 0.63–0.82), with an area under the HSROC curve of 0.81 (95% CI, 0.77–0.84) (Figure 2). The corresponding positive likelihood ratio was 2.8 (95% CI, 2.0–4.1) and the negative likelihood ratio 0.33 (95% CI, 0.21–0.54), resulting in a diagnostic odds ratio of 9 (95% CI, 4–18). We found substantial heterogeneity (*I*^2^ = 98%, *p* < 0.001) (Supplementary Figure S2), but the threshold effects were minimal (2% of heterogeneity), indicating variability largely due to factors other than differing score cut-offs.

**Figure 3:**
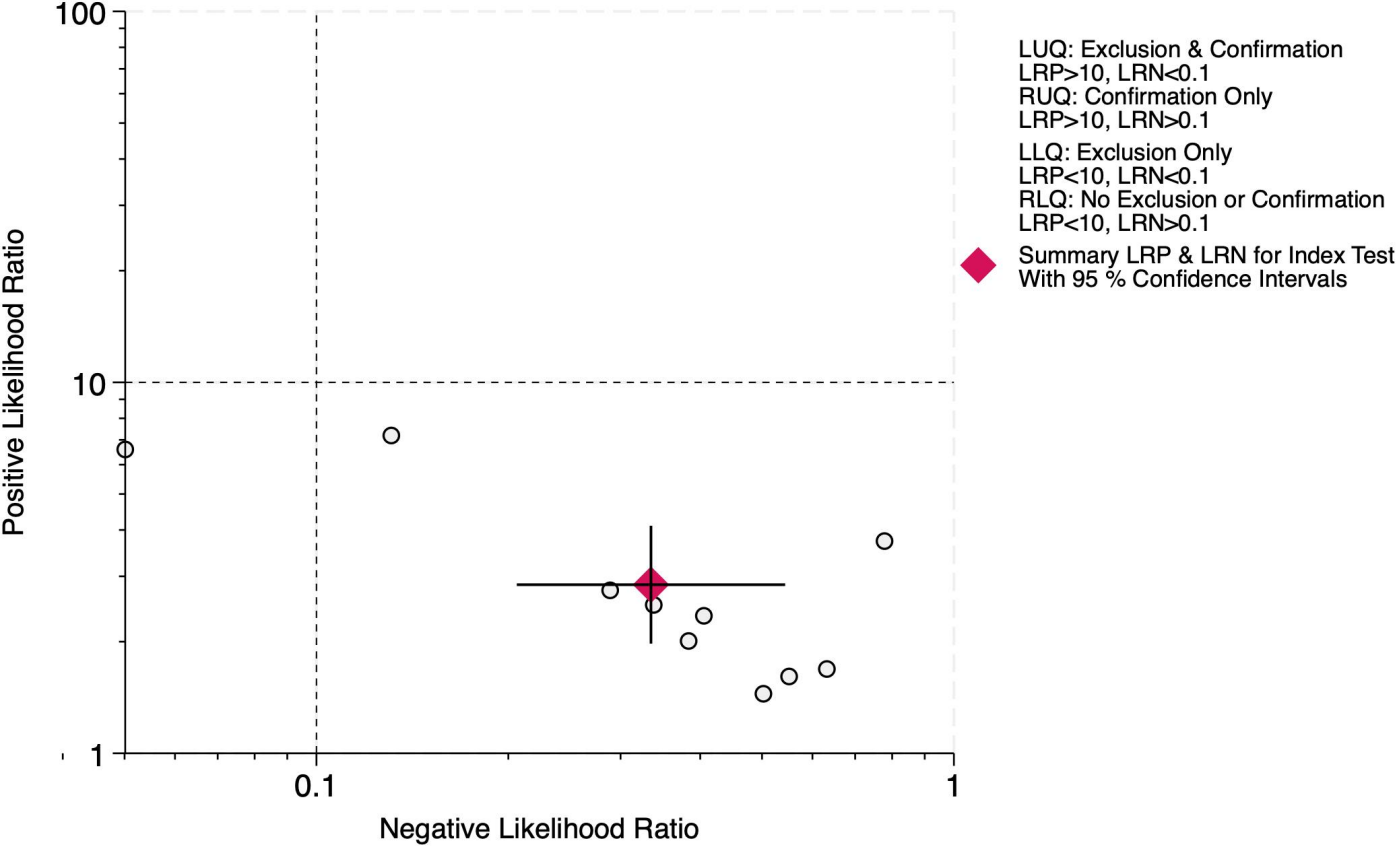
Likelihood ratio scattergram for diagnostic accuracy of lung ultrasound scores for bronchopulmonary dysplasia at first three days of life.

**Figure 4:**
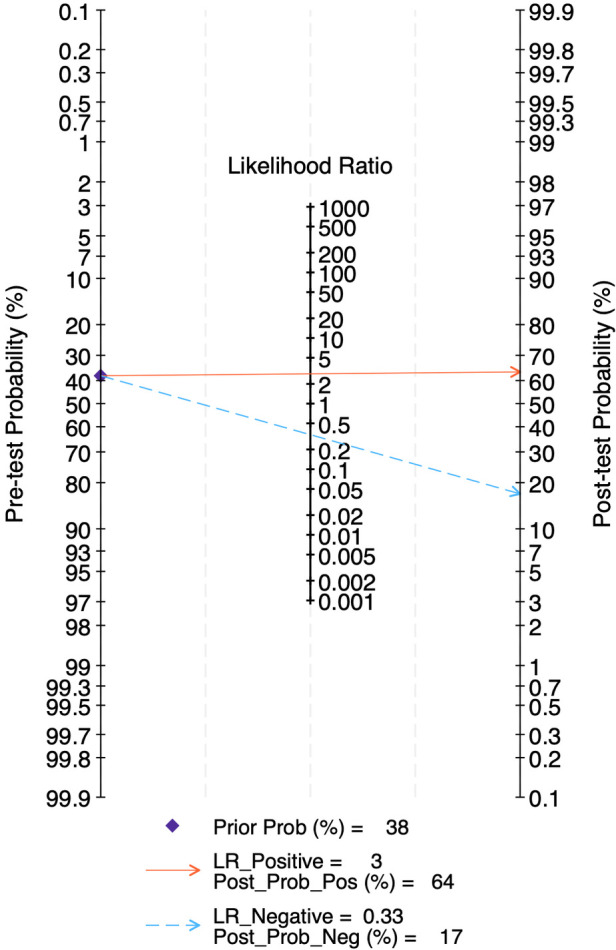
Fagan's nomogram for diagnostic accuracy of lung ultrasound scores for bronchopulmonary dysplasia at first three days of life.

The LR scattergram (Figure 3) showed the overall pooled estimate in the right lower quadrant indicating that the LUS score should not be used for BPD confirmation or exclusion on the first three days of life. At a pre-test probability of 38%, the pooled positive likelihood ratio of 3.0 raises the post-test probability of BPD to 64%, while the negative likelihood ratio of 0.33 reduces it to 17%, as illustrated by the Fagan nomogram (Figure 4). Deek's test showed absence of publication bias (*p* = 0.54) with a symmetrical funnel plot (Supplementary Figure S3). Subgroup analysis was tried based on gestational age (<28 weeks vs. ≥28 weeks). However, there were fewer studies under <28 weeks and hence, subgroup results was incalculable. For ≥28 weeks subgroup, the pooled sensitivity and specificity was 0.69 [95%CI: 0.54–0.81] and 0.70 [95%CI: 0.57–0.81] with AUROC of 0.75 [95%CI: 0.71–0.79).

### Diagnostic accuracy of LUS score for BPD on day 7

**Figure 5:**
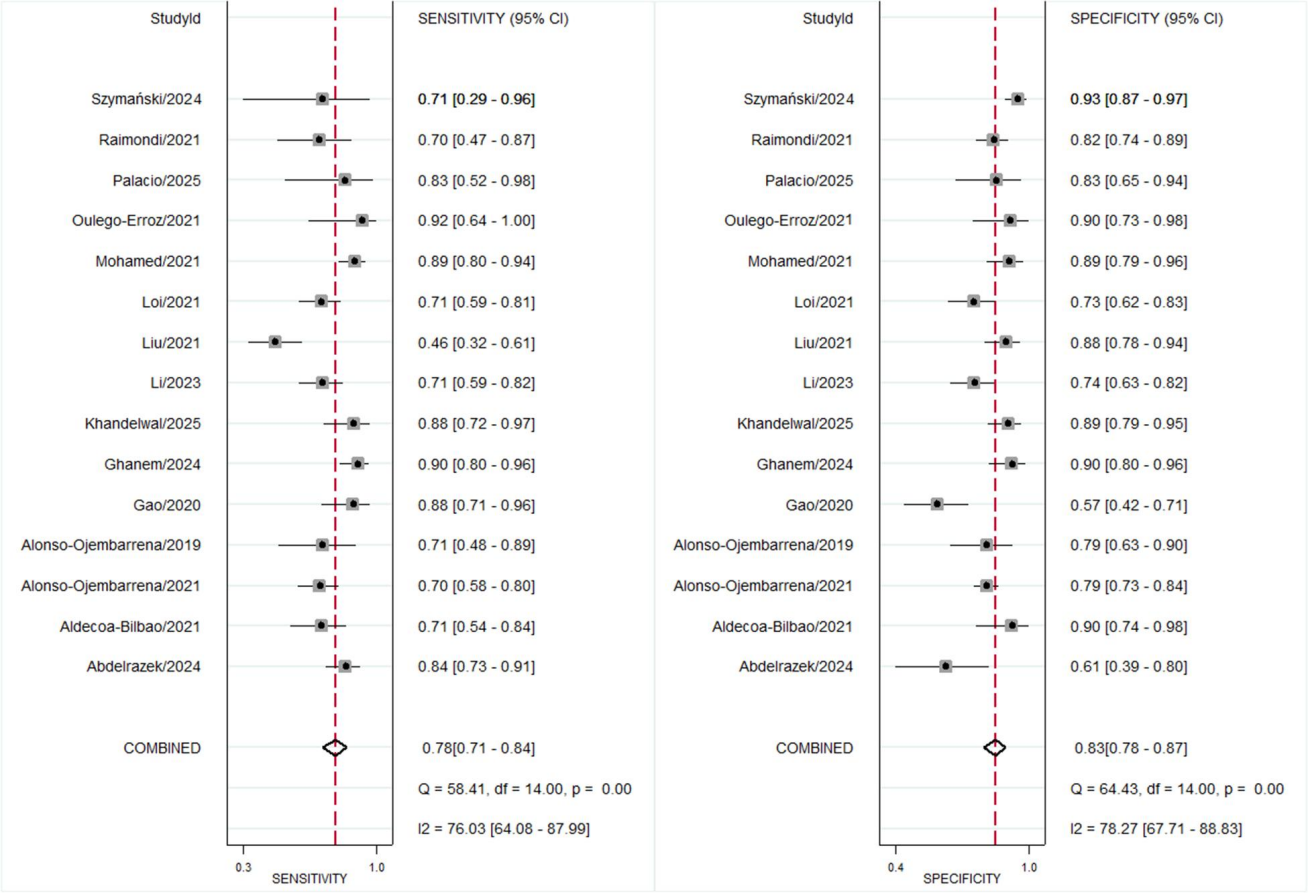
Forest plot for diagnostic accuracy of lung ultrasound scores for bronchopulmonary dysplasia at postnatal day 7.

**Figure 6:**
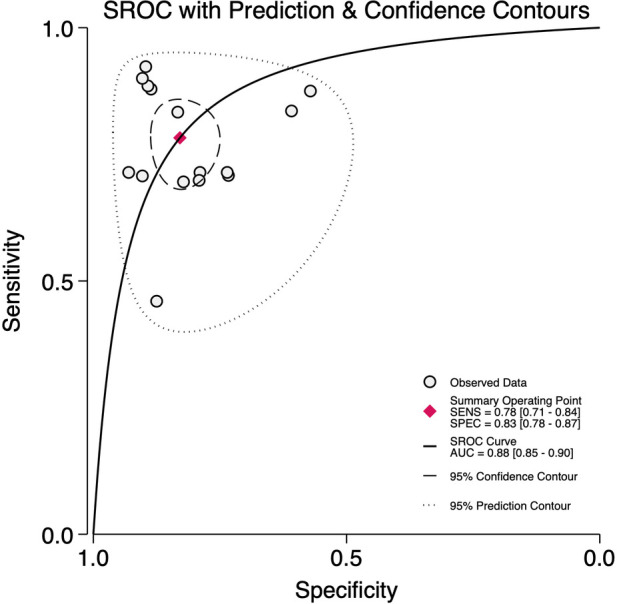
Summary receiver operator characteristics curve plot for diagnostic accuracy of lung ultrasound scores for bronchopulmonary dysplasia at postnatal day 7.

Across 15 studies encompassing 670 BPD-positive and 1,120 BPD-negative preterm infants at 7 days of life, the pre-test probability of disease was 37%. The bivariate meta-analysis (Figure 5) yielded a pooled sensitivity of 0.78 (95% CI, 0.71–0.84) and a pooled specificity of 0.83 (95% CI, 0.78–0.87), with an HSROC area of 0.88 (95% CI, 0.85–0.90) (Figure 6). The positive likelihood ratio was 4.6 (95% CI, 3.4–6.2) and the negative likelihood ratio 0.26 (95% CI, 0.19–0.35), corresponding to a diagnostic odds ratio of 17 (95% CI, 10–29). There remained substantial heterogeneity (*I*^2^ = 88%, *p* < 0.001), although threshold effects were negligible (Supplementary Figure S4).

**Figure 7:**
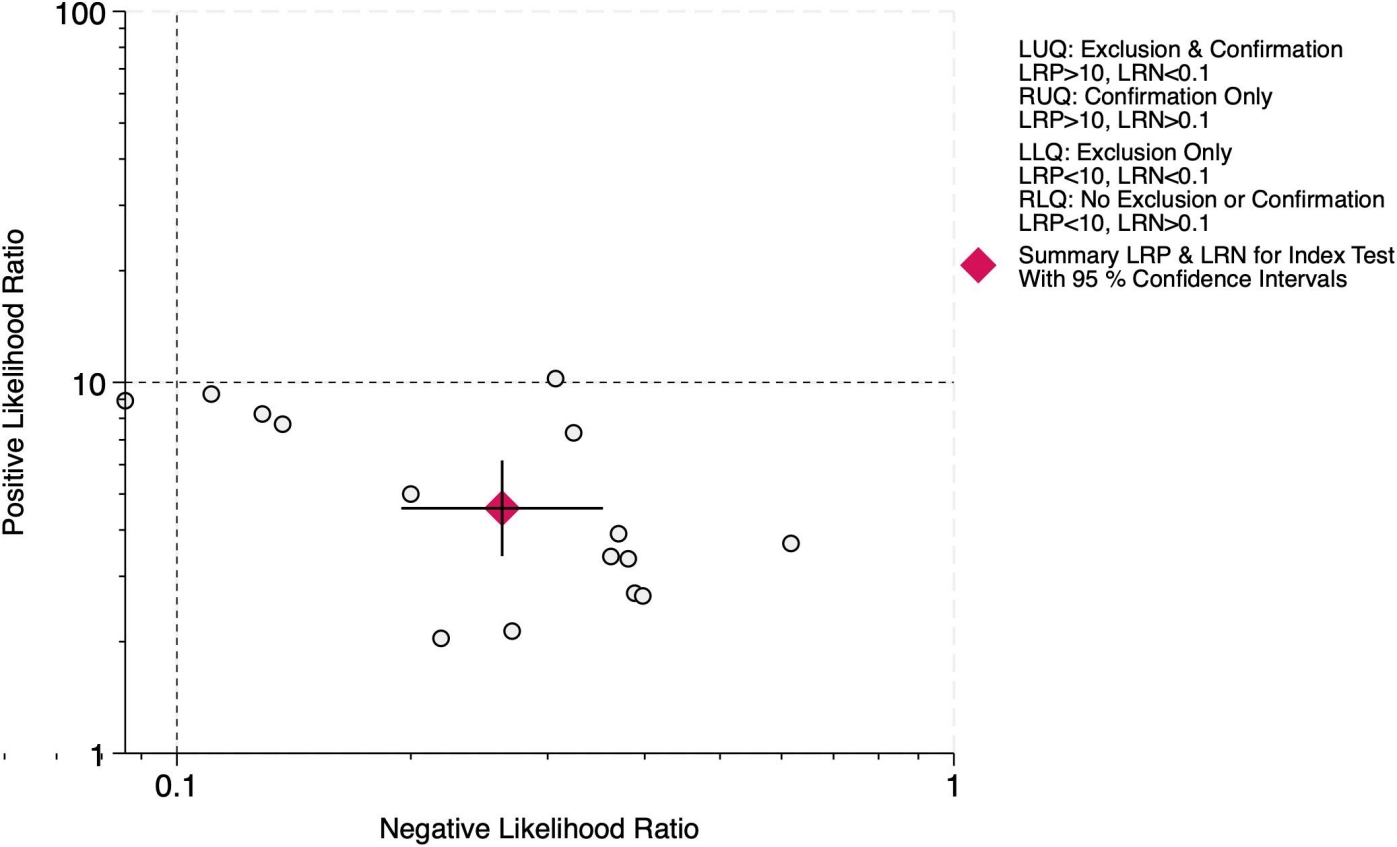
Likelihood ratio scattergram for diagnostic accuracy of lung ultrasound scores for bronchopulmonary dysplasia at postnatal day 7.

**Figure 8:**
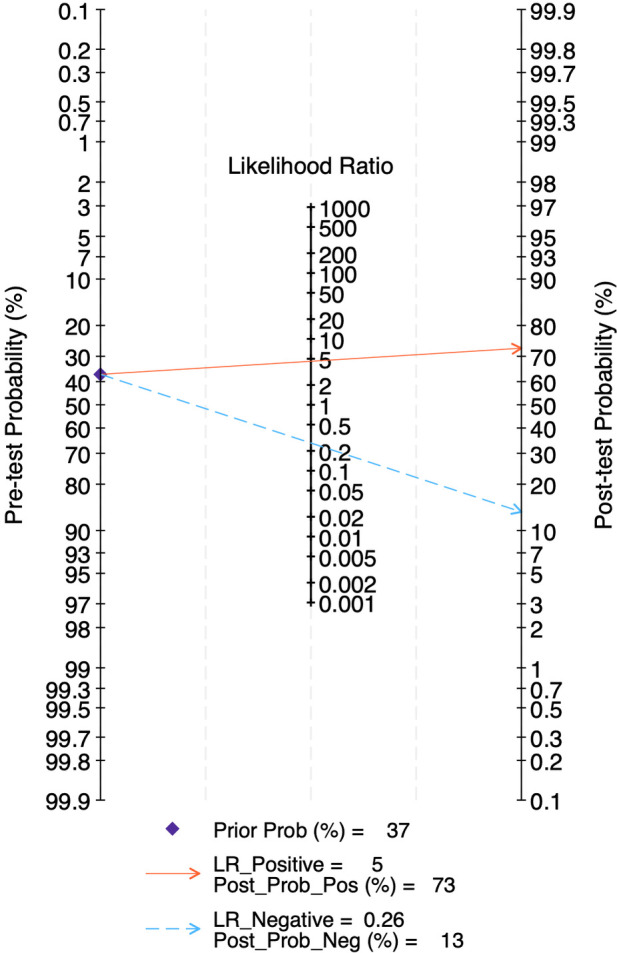
Fagan's nomogram for diagnostic accuracy of lung ultrasound scores for bronchopulmonary dysplasia at postnatal day 7.

The LR scattergram (Figure 7) showed the pooled estimate in the right lower quadrant indicating that the LUS score should not be used for confirmation or exclusion on day 7. At a pre-test probability of 37%, the pooled positive likelihood ratio of 5.0 raises the post-test probability of BPD to 73%, while the negative likelihood ratio of 0.36 reduces it to 13%, as illustrated by the Fagan nomogram (Figure 8). Deek's test results showed absence of publication bias (*p* = 0.44) with a symmetrical funnel plot (Supplementary Figure S5).

Subgroup analysis was tried based on gestational age (<28 weeks vs. ≥28 weeks). For <28 weeks, the pooled sensitivity and specificity was 0.84 [95%CI: 0.75–0.91] and 0.80 [95%CI: 0.64–0.90] respectively with AUROC of 0.89 [95%CI: 0.86–0.92). For ≥28 weeks subgroup, the pooled sensitivity and specificity was 0.74 [95%CI: 0.65–0.81] and 0.84 [95%CI: 0.79–0.88] with AUROC of 0.86 [95%CI: 0.83–0.89).

### Diagnostic accuracy of LUS score for BPD on day 14

**Figure 9:**
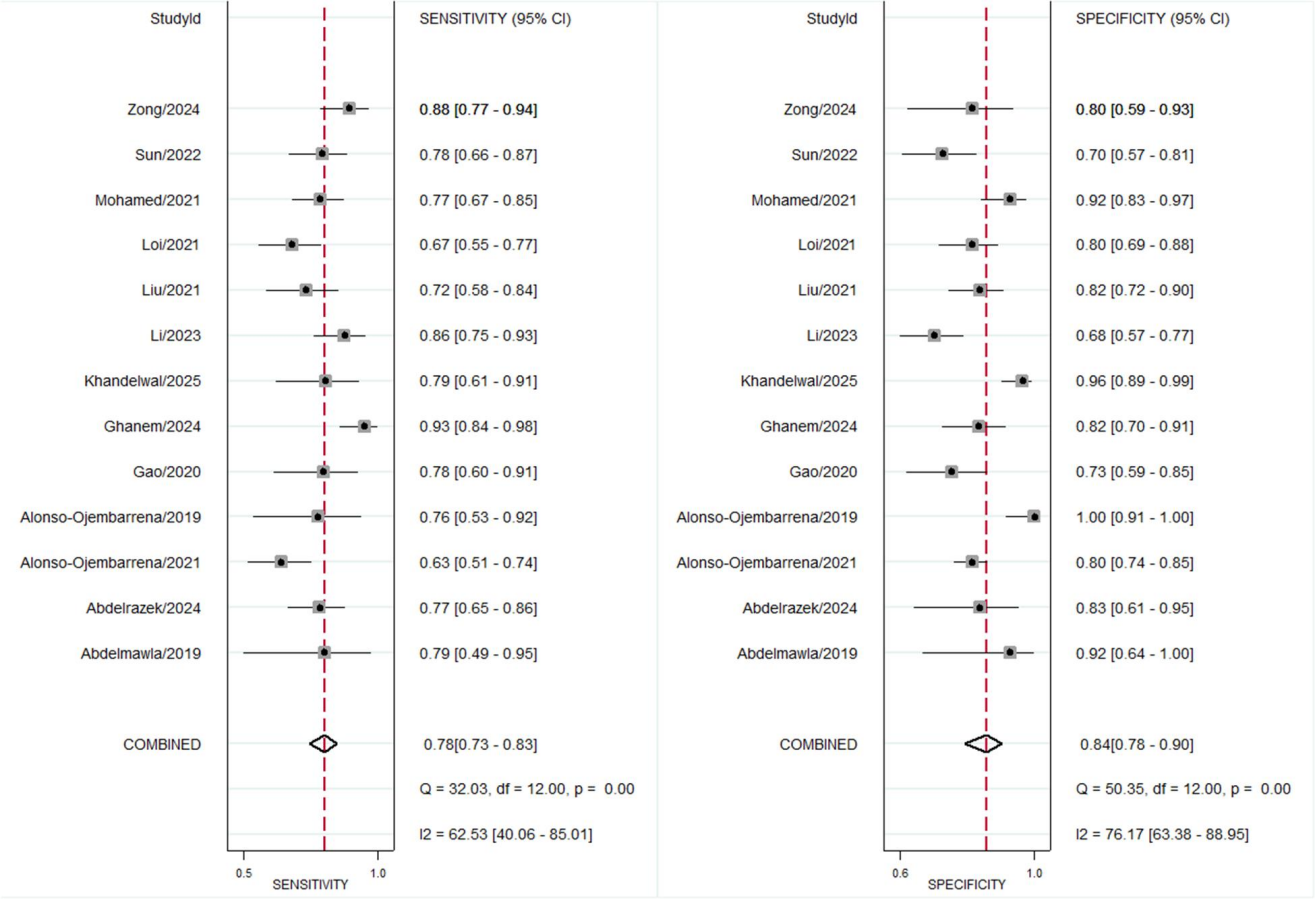
Forest plot for diagnostic accuracy of lung ultrasound scores for bronchopulmonary dysplasia at postnatal day 14.

**Figure 10:**
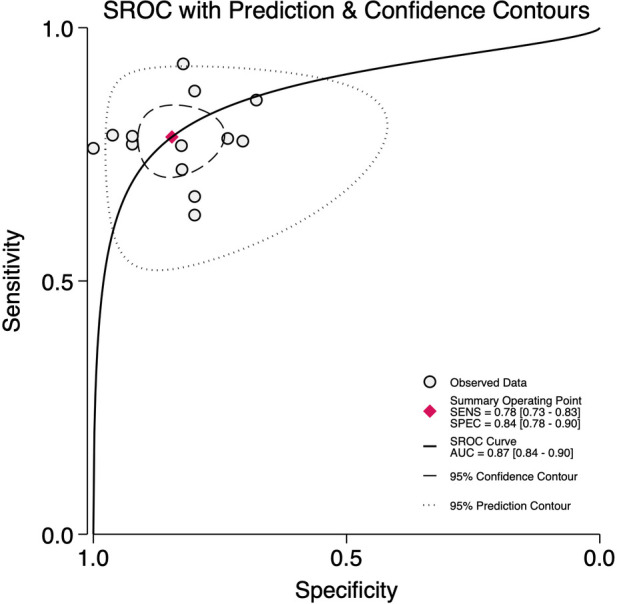
Summary receiver operator characteristics curve plot for diagnostic accuracy of lung ultrasound scores for bronchopulmonary dysplasia at postnatal day 14.

Across thirteen studies including 719 BPD-positive and 882 BPD-negative preterm infants at 14 days of life, the pre-test probability of disease was 45%. The pooled sensitivity was 0.78 (95% CI, 0.73–0.83) and the pooled specificity 0.84 (95% CI, 0.78–0.90) (Figure 9), with an area under the HSROC curve of 0.87 (95% CI, 0.84–0.90) (Figure 10). The positive likelihood ratio was 5.1 (95% CI, 3.5–7.4) and the negative likelihood ratio 0.26 (95% CI, 0.20–0.32), yielding a diagnostic odds ratio of 20 (95% CI, 12–33). Substantial heterogeneity was observed (*I*^2^ = 88%, *p* < 0.001), with minimal threshold effects (Supplementary Figure S6).

**Figure 11:**
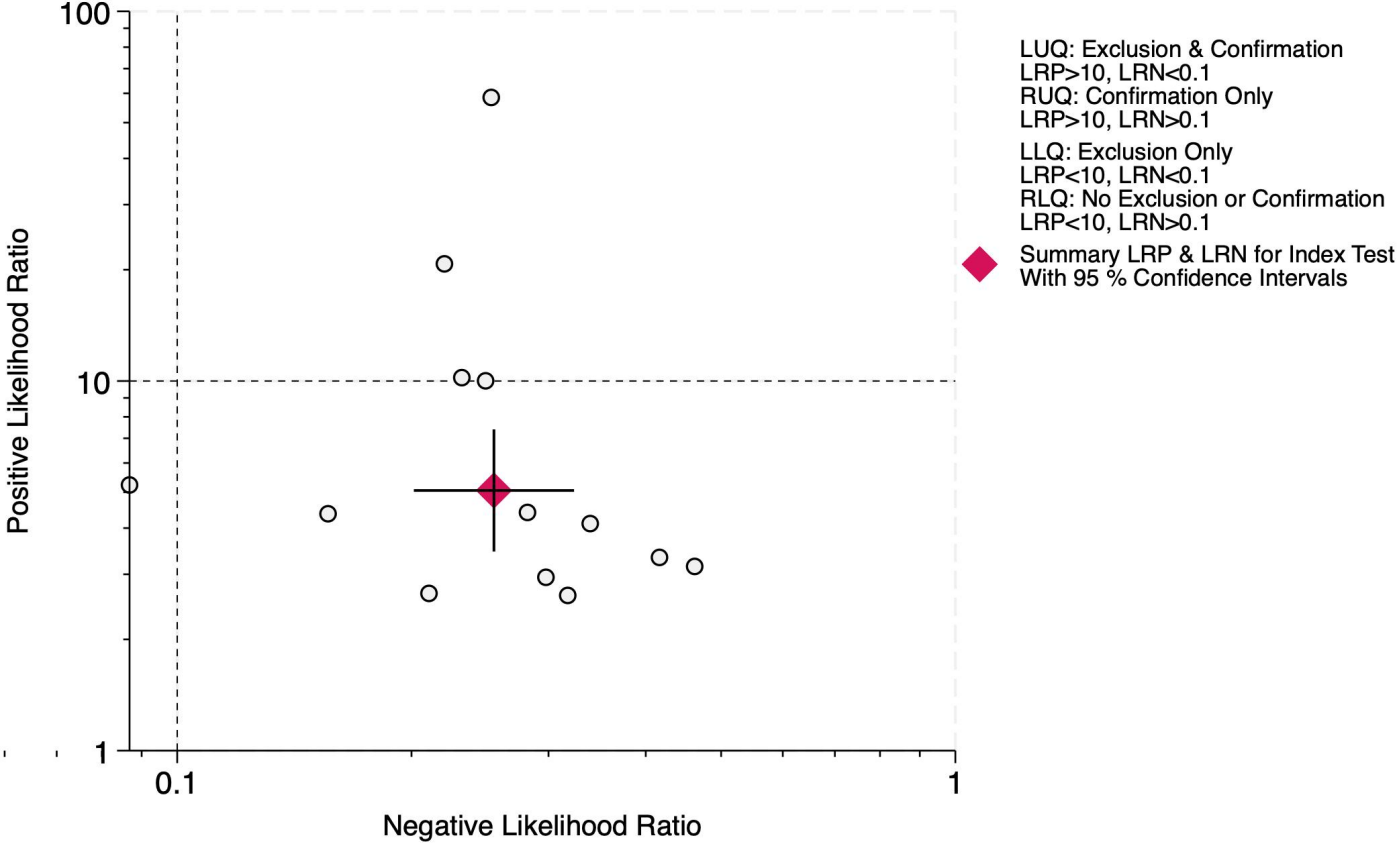
Likelihood ratio scattergram for diagnostic accuracy of lung ultrasound scores for bronchopulmonary dysplasia at postnatal day 14.

**Figure 12:**
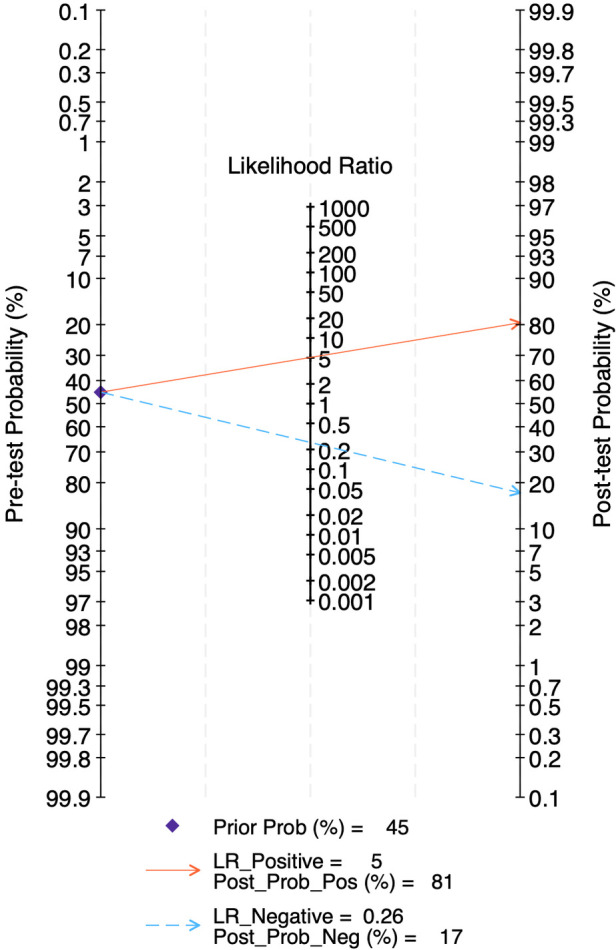
Fagan's nomogram for diagnostic accuracy of lung ultrasound scores for bronchopulmonary dysplasia at postnatal day 14.

The LR scattergram (Figure 11) showed the pooled estimate in the right lower quadrant indicating that the LUS score should not be used for confirmation or exclusion on day 14. At a pre-test probability of 45%, the pooled positive likelihood ratio of 5.0 raises the post-test probability of BPD to 81%, while the negative likelihood ratio of 0.26 reduces it to 17%, as illustrated by the Fagan nomogram (Figure 12). Deek's test results indicated potential publication bias (*p* = 0.09) with a slightly asymmetrical funnel plot (Supplementary Figure S7).

Subgroup analysis was tried based on gestational age (<28 weeks vs. ≥28 weeks). For <28 weeks, the pooled sensitivity and specificity was 0.82 [95%CI: 0.72–0.88] and 0.83 [95%CI: 0.77–0.88] respectively with AUROC of 0.89 [95%CI: 0.86–0.91). For ≥28 weeks subgroup, the pooled sensitivity and specificity was 0.76 [95%CI: 0.69–0.81] and 0.86 [95%CI: 0.73–0.93] with AUROC of 0.82 [95%CI: 0.78–0.85).

### Diagnostic accuracy of LUS score for BPD on days 21 & 28

**Figure 13:**
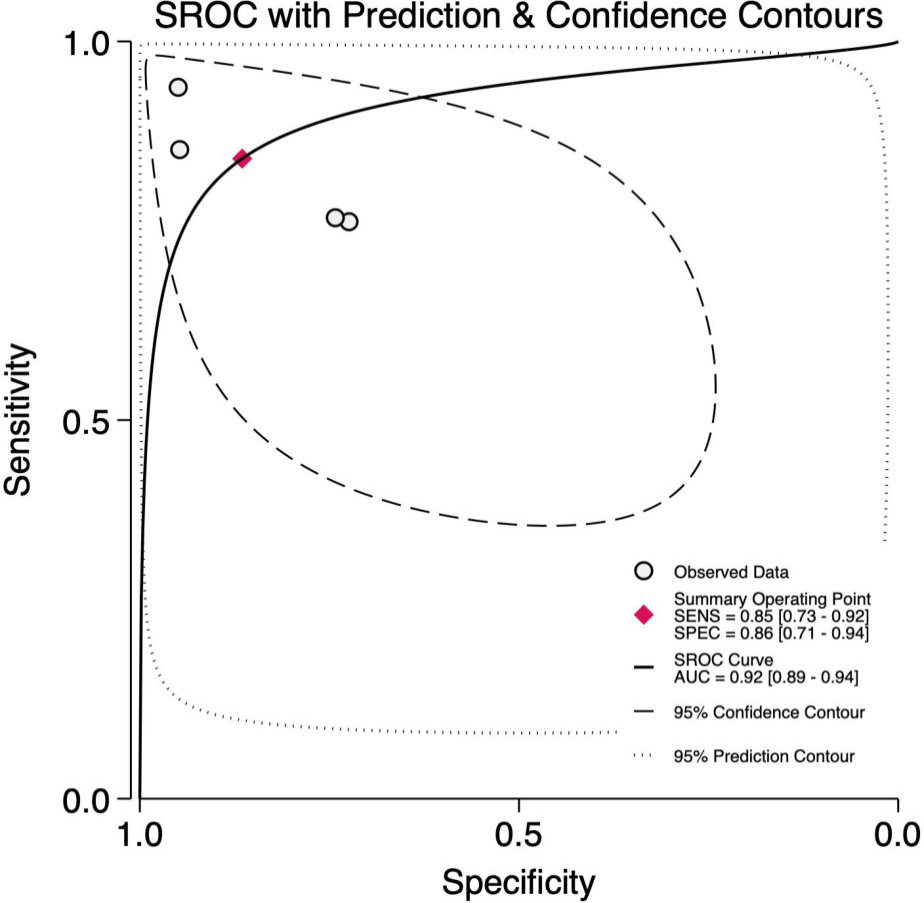
Summary receiver operator characteristics curve plot for diagnostic accuracy of lung ultrasound scores for bronchopulmonary dysplasia at postnatal day 21.

At 21 days of life, data from four studies including 190 BPD-positive and 429 BPD-negative infants yielded a pre-test probability of 31%. The pooled sensitivity was 0.85 (95% CI, 0.73–0.92) and specificity 0.86 (95% CI, 0.71–0.94), with an HSROC area of 0.92 (95% CI, 0.89–0.94) (Figure 13). The positive likelihood ratio was 6.3 (95% CI, 2.5–15.5) and the negative likelihood ratio 0.18 (95% CI, 0.09–0.36), corresponding to a diagnostic odds ratio of 35 (95% CI, 8–163). Heterogeneity was negligible (*I*^2^ = 0%, *p* = 0.32), and threshold effects accounted for virtually all variability, reflecting consistent test performance at this time point (Supplementary Figure S8).

**Figure 14:**
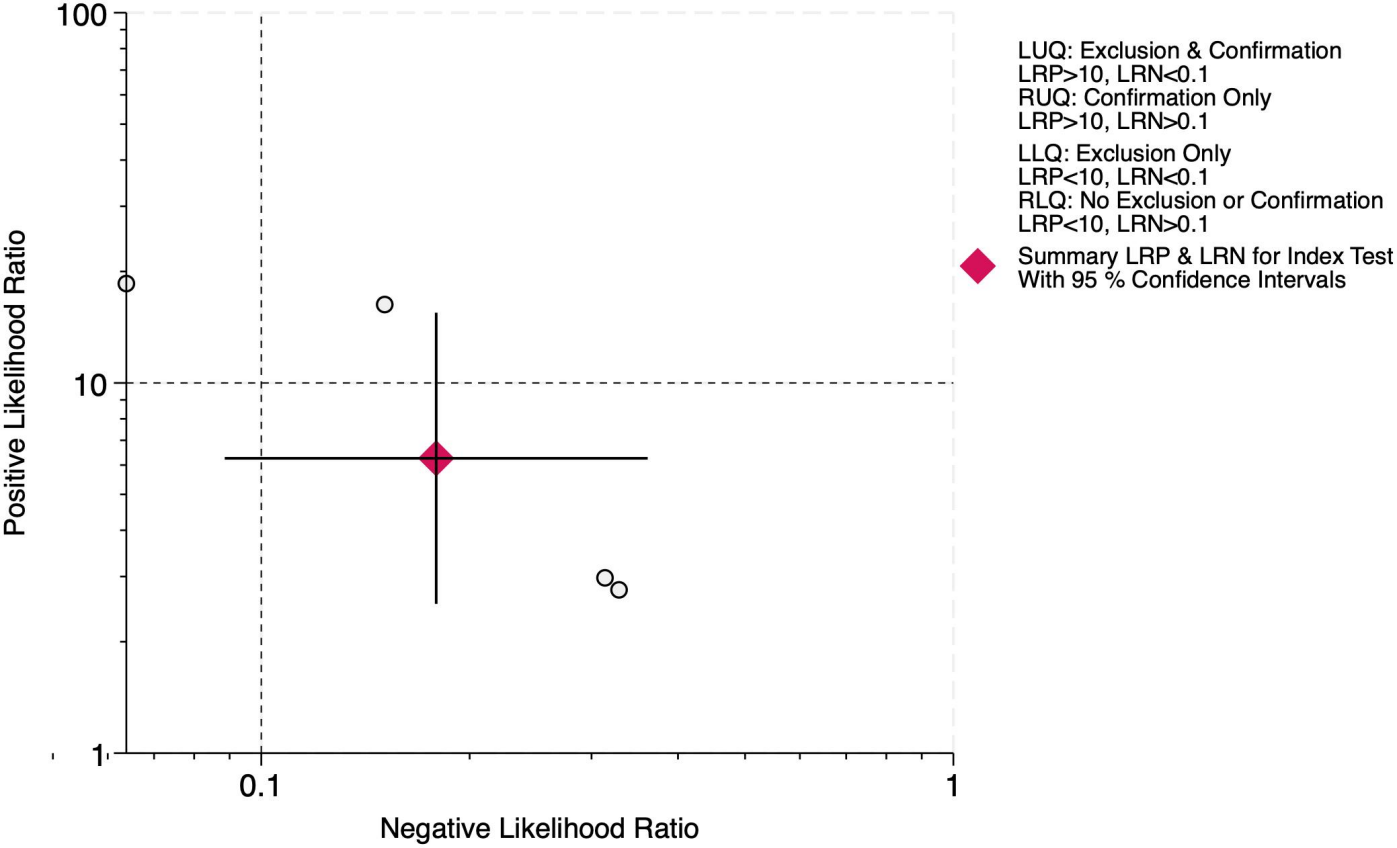
Likelihood ratio scattergram for diagnostic accuracy of lung ultrasound scores for bronchopulmonary dysplasia at postnatal day 21.

**Figure 15:**
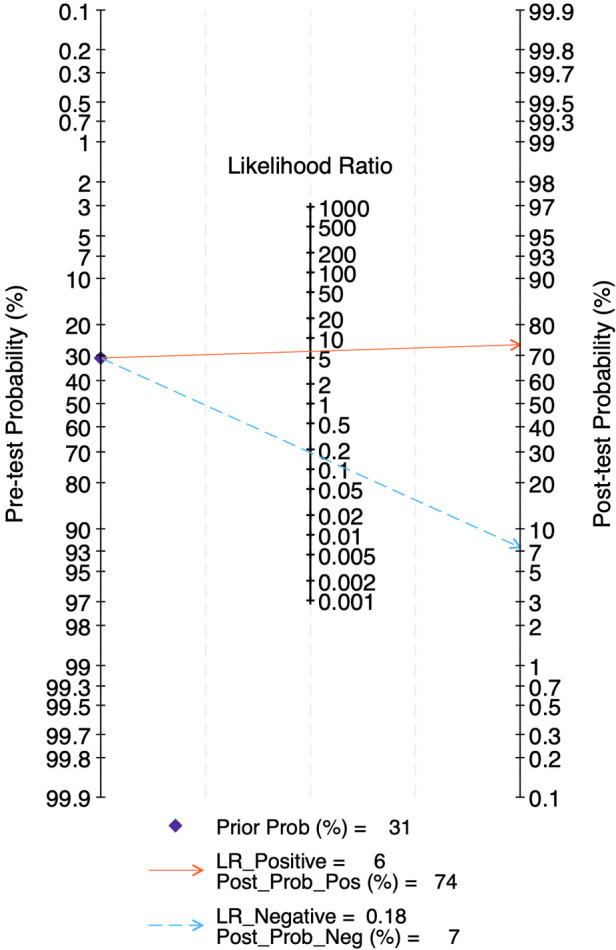
Fagan's nomogram for diagnostic accuracy of lung ultrasound scores for bronchopulmonary dysplasia at postnatal day 21.

The LR scattergram (Figure 14) showed the pooled estimate in the right lower quadrant indicating that the LUS score should not be used for confirmation or exclusion on day 21, but the confidence interval extends to left lower quadrant indicating the possibility of good rule-out capacity at day 21. At a pre-test probability of 31%, the pooled positive likelihood ratio of 6.0 raises the post-test probability of BPD to 74%, while the negative likelihood ratio of 0.18 reduces it to 7%, as illustrated by the Fagan nomogram (Figure 15). Deek's test showed absence of publication bias (*p* = 0.22) with a symmetrical funnel plot (Supplementary Figure S9). Subgroup analysis was tried based on gestational age (<28 weeks vs. ≥28 weeks). However, there were fewer studies under <28 weeks and hence, subgroup results was incalculable. For ≥28 weeks subgroup, the pooled sensitivity and specificity was 0.85 [95%CI: 0.73–0.92] and 0.86 [95%CI: 0.71–0.94] with AUROC of 0.92 [95%CI: 0.89–0.94).

Three studies reported the diagnostic accuracy of LUS score for BPD at day 28. We required a minimum of four studies to perform a diagnostic accuracy meta-analysis; therefore, we could not pool the estimates and report the final diagnostic accuracy measures at 28 days of life.

## Discussion

Our results show that semiquantitative LUS scoring achieves increasingly robust BPD diagnostic accuracy in preterm infants as postnatal age advances. Within the first three days of life, across ten studies and more than 1,300 infants, LUS scores yielded moderate sensitivity and specificity (approximately 0.75 and 0.74, respectively) with an AUROC of 0.81. By day seven, with data from 15 studies, both sensitivity and specificity improved to approximately 0.78 and 0.83, and the AUROC rose to 0.88. At 14 days of age, the pooled estimates remained stable (sensitivity ∼0.78, specificity ∼0.84, AUROC 0.87). However, by day 21 albeit in a smaller number of studies, LUS performance peaked, achieving a sensitivity of 0.85 and a specificity of 0.86 with an AUROC of 0.92. Across all time points, the positive likelihood ratios increased from roughly 2.8 in the first three days to over 6 by day 21, while negative likelihood ratios decreased from about 0.33–0.18, reflecting both better rule-in and rule-out capabilities over time. The heterogeneity was substantial early on but diminished markedly by day 21, and the threshold effects were minimal at every interval, indicating that the variability stemmed more from study-level factors than from divergent cut-offs. Collectively, these results suggest that while LUS scoring has modest accuracy immediately after birth, its clinical utility gets stronger by the third week of life, approaching the diagnostic reliability of conventional criteria without the need for ionizing radiation. Our findings align with and extend earlier narrative reviews and single-centre cohort studies that identified LUS as a promising bedside tool for BPD assessment (43). Initial investigations often reported sensitivities and specificities in the range of 0.70–0.80 for LUS examinations performed in the first week of life, mirroring our pooled estimates at days three and seven. However, many of those early reports were underpowered and lacked formal meta-analytic synthesis. Two prior systematic reviews were limited to seven and ten studies and focused predominantly on 36-week PMA assessments that found pooled accuracy measures that were similar to our day-14 results, but failed to capture the dynamic progression of test performance (17, 44). By contrast, our time-stratified approach clarifies how LUS scores evolve in parallel with the natural history of pulmonary parenchymal changes.

Some authors have suggested that the accuracy of LUS scores may plateau after the first week. However, our analysis reveals that the diagnostic performance of LUS continues to improve through the third week of life, a finding supported by smaller cohorts that have emphasized the value of serial imaging (23, 28, 31). By contrast, studies that have applied static LUS protocols at a single time point and reported wide ranges of accuracy often reflect heterogeneity in the scanning technique and scoring thresholds (44). By aggregating data across standardized zones and semiquantitative scales, and by isolating time-specific performance values, our meta-analysis results demonstrate that the timing of ultrasound is a critical determinant of accuracy, an insight only hinted at in the previous literature (45).

The exceptionally high AUROC of 0.92 at day 21 demonstrates that LUS scoring can rival, and in some respects exceed, the diagnostic precision of the standard BDP diagnostic criteria (chest radiography and oxygen-dependency), which themselves exhibit interobserver variability and require specialized transport (46). The superior late-timepoint accuracy of LUS scoring challenges the prevailing paradigm that considers radiography indispensable for BPD diagnosis, suggesting instead that bedside ultrasound may serve as a primary diagnostic modality, particularly when repeated assessments are clinically indicated. By systematically comparing our pooled estimates to the reference-standard performance reported in historical cohorts, we highlighted the incremental gains achieved by LUS scoring and established a benchmark for future comparative studies.

The progressive improvement in diagnostic accuracy of LUS scoring observed over the first three weeks of life likely reflects the evolving pathophysiology of BPD. In the immediate postnatal period, preterm lungs lack surfactant, retain lung fluid, and present variable degrees of parenchymal atelectasis, yielding ultrasound findings such as coalescent B-lines and subpleural consolidations that overlap with symptoms of transient respiratory distress syndrome (47). As infants age, however, the pathologic hallmarks of BPD, including impaired alveolarization, interstitial fibrosis, and heterogeneous aeration, become more pronounced and detectable by semiquantitative ultrasound (48). By day 7, partial clearance of lung fluid unmasks persistent interstitial changes, improving the signal-to-noise ratio for B-line quantification. By two to three weeks, the fibrotic remodelling and airway injury underlying moderate to severe BPD generate distinct pleural irregularities and focal consolidations that allow for reliable scoring (49). These dynamic physio-pathological transitions explain why LUS scores discriminate poorly in the first days of life but achieve near-optimal sensitivity and specificity by day 21. Moreover, the minimal threshold effects across studies indicate that this temporal enhancement is driven by genuine changes in lung structure rather than variations in scoring cut-offs. In this comprehensive analysis, we synthesized data from 22 studies and over 2,000 preterm infants, applying rigorous bivariate modelling to jointly estimate sensitivity and specificity at multiple clinically relevant time points. The inclusion of prospective cohorts from diverse geographic settings enhanced the external validity of our results. Moreover, the formal risk-of-bias assessments and sensitivity analyses bolstered confidence in the pooled estimates. By stratifying performance by postnatal age, we uncovered the time-dependent utility of LUS, a nuance overlooked in prior reviews. However, limitations merit consideration. The substantial heterogeneity in early time-point analyses reflects variation in LUS protocols, operator training, and timing of imaging examinations, underscoring the need for standardized scanning and scoring guidelines. Although threshold effects were minimal, a small number of studies at later intervals particularly at day 21 may have overestimated the LUS scoring performance due to publication bias or selective reporting. In addition, most data are derived from tertiary NICUs with experienced sonographers, limiting generalizability to centres with less ultrasound expertise. In addition, the variability in BPD definitions and reliance on oxygen-dependency criteria as reference standards may have introduced misclassifications, suggesting that pooled accuracy metrics could differ if more stringent physiological or long-term pulmonary function measures are applied. Our findings demonstrate that LUS scoring may be a valuable, non-invasive adjunct in the diagnostic toolkit against BPD. In the first week of life, when LUS score accuracy is modest, the technique may serve primarily as an early screening tool to identify infants warranting closer monitoring or adjunctive assessments. By two weeks of age, the high likelihood ratios support its use in guiding clinical decisions such as timing of corticosteroid therapy, optimization of ventilator settings, and discharge planning potentially reducing reliance on chest radiography and minimizing radiation exposure. The portability and repeatability of LUS enable serial assessments at the bedside, fostering dynamic risk stratification without transporting critically ill neonates. In addition, in settings with limited radiology resources, LUS may offer a pragmatic alternative for BPD evaluation, provided adequate training and quality assurance measures are in place. Integration of standardized LUS protocols into NICU workflows could enhance early identification of infants at highest risk for chronic lung disease and facilitate timely, evidence-based interventions. To translate these promising accuracy estimates into practice, future studies should prioritize standardization of LUS protocols, including agreement on the number and anatomical location of scanning zones, semiquantitative scoring thresholds, and optimal timing of examinations. Multi-centre trials comparing LUS-guided management algorithms against standard of care are needed to determine whether ultrasound surveillance can improve clinical outcomes, such as rates of home oxygen therapy, rehospitalization, and neurodevelopmental sequelae. Researchers should also explore the learning curve for LUS proficiency among various provider types and assess inter-observer reproducibility in real-world settings. Cost-effectiveness analyses will help quantify resource savings from reduced radiography and short NICU stays. Finally, long-term follow-up studies correlating early LUS scores with pulmonary function tests in childhood are needed to validate the prognostic value of ultrasound markers and refine risk-stratification models for BPD.

## Conclusion

Semiquantitative LUS scoring offers a radiation-free, bedside diagnostic modality whose accuracy for BPD improves markedly with postnatal age, reaching optimal performance by the third week of life. While early assessments provide moderate discrimination, later timepoints yield high sensitivity and specificity, supporting LUS scoring as both a confirmatory and exclusionary tool. Adoption of standardized protocols and integration into clinical pathways hold the promise of enhancing early detection, guiding targeted interventions, and ultimately improving respiratory outcomes for vulnerable preterm infants.

## Data Availability

The datasets presented in this study can be found in online repositories. The names of the repository/repositories and accession number(s) can be found in the article/Supplementary Material.

## Supplementary material

Supplementary Figure S1 PRISMA flowchart.

Supplementary Figure S2 Bivariate box plot for lung ultrasound scores for bronchopulmonary dysplasia at first three days of life.

Supplementary Figure S3 Deek's funnel plot for lung ultrasound scores for bronchopulmonary dysplasia at first three days of life.

Supplementary Figure S4 Bivariate box plot for lung ultrasound scores for bronchopulmonary dysplasia at postnatal day 7.

Supplementary Figure S5 Deek's funnel plot for lung ultrasound scores for bronchopulmonary dysplasia at postnatal day 7.

Supplementary Figure S6 Bivariate box plot for lung ultrasound scores for bronchopulmonary dysplasia at postnatal day 14.

Supplementary Figure S7 Deek's funnel plot for lung ultrasound scores for bronchopulmonary dysplasia at postnatal day 14.

Supplementary Figure S8 Bivariate box plot for lung ultrasound scores for bronchopulmonary dysplasia at postnatal day 21.

Supplementary Figure S9 Deek's funnel plot for lung ultrasound scores for bronchopulmonary dysplasia at postnatal day 21.

## References

[1] Collaco JM McGrath-Morrow SA. Bronchopulmonary dysplasia as a determinant of respiratory outcomes in adult life. Pediatr Pulmonol. (2021) 56:3464–71. 10.1002/ppul.25301 33730436 PMC8446084

[2] Sahni M Mowes AK. Bronchopulmonary dysplasia. StatPearls. Treasure Island, FL: StatPearls Publishing (2025). Available online at: http://www.ncbi.nlm.nih.gov/books/NBK539879/ (Accessed August 6, 2025) 30969701

[3] Kennedy KA Cotten CM Watterberg KL Carlo WA. Prevention and management of bronchopulmonary dysplasia: lessons learned from the neonatal research network. Semin Perinatol. (2016) 40:348–55. 10.1053/j.semperi.2016.05.010 27742002 PMC5279709

[4] Li W Wang Y Song J Zhang C Xu Y Xu F Association between bronchopulmonary dysplasia and death or neurodevelopmental impairment at 3 years in preterm infants without severe brain injury. Front Neurol. (2023) 14:1292372. 10.3389/fneur.2023.1292372 38033771 PMC10684711

[5] Jensen EA Schmidt B. Epidemiology of bronchopulmonary dysplasia. Birth Defects Res A Clin Mol Teratol. (2014) 100:145–57. 10.1002/bdra.23235 24639412 PMC8604158

[6] Sucasas-Alonso A Pértega-Díaz S Balboa-Barreiro V García-Muñoz Rodrigo F Avila-Alvarez A. Prediction of bronchopulmonary dysplasia in very preterm infants: competitive risk model nomogram. Front Pediatr. (2024) 12:1335891. 10.3389/fped.2024.1335891 38445078 PMC10912561

[7] Wielpütz MO Heußel CP Herth FJF Kauczor H-U. Radiological diagnosis in lung disease: factoring treatment options into the choice of diagnostic modality. Dtsch Arztebl Int. (2014) 111:181–7. 10.3238/arztebl.2014.0181 24698073 PMC3977441

[8] Akram S Chowdhury YS. Radiation exposure of medical imaging. StatPearls. Treasure Island, FL: StatPearls Publishing (2025). Available online at: http://www.ncbi.nlm.nih.gov/books/NBK565909/ (Accessed August 6, 2025) 33351446

[9] Emmerson BR Young M. Radiology patient safety and communication. StatPearls. Treasure Island, FL: StatPearls Publishing (2025). Available online at: http://www.ncbi.nlm.nih.gov/books/NBK567713/ (Accessed August 6, 2025) 33620790

[10] Gilfillan M Bhandari A Bhandari V. Diagnosis and management of bronchopulmonary dysplasia. Br Med J. (2021) 375:n1974. 10.1136/bmj.n1974 34670756

[11] Saraogi A. Lung ultrasound: present and future. Lung India. (2015) 32:250–7. 10.4103/0970-2113.156245 25983411 PMC4429387

[12] Iovine E Nenna R Bloise S La Regina DP Pepino D Petrarca L Lung ultrasound: its findings and new applications in neonatology and pediatric diseases. Diagnostics (Basel). (2021) 11:652. 10.3390/diagnostics11040652 33916882 PMC8066390

[13] Deng Q Zhang Y Wang H Chen L Yang Z Peng Z Semiquantitative lung ultrasound scores in the evaluation and follow-up of critically ill patients with COVID-19: a single-center study. Acad Radiol. (2020) 27:1363–72. 10.1016/j.acra.2020.07.002 32713715 PMC7359788

[14] Baciarello M Bonetti A Vetrugno L Saturno F Nouvenne A Bellini V Is lung ultrasound score a useful tool to monitoring and handling moderate and severe COVID-19 patients in the general ward? An observational pilot study. J Clin Monit Comput. (2022) 36:785–93. 10.1007/s10877-021-00709-w 33948780 PMC8096129

[15] Abdelrazek AA Kamel SM Elbakry AAE Elmazzahy EA. Lung ultrasound in early prediction of bronchopulmonary dysplasia in pre-term babies. J Ultrasound. (2024) 27:653–62. 10.1007/s40477-024-00913-9 38907789 PMC11333650

[16] Zong H Huang Z Zhao J Lin B Fu Y Lin Y The value of lung ultrasound score in neonatology. Front Pediatr. (2022) 10:791664. 10.3389/fped.2022.791664 35633958 PMC9130655

[17] Zhang X Yang X Li Y. Lung ultrasound score for prediction of bronchopulmonary dysplasia in newborns: a meta-analysis. Technol Health Care. (2025) 33:235–45. 10.3233/THC-240832 39302397

[18] Pezza L Alonso-Ojembarrena A Elsayed Y Yousef N Vedovelli L Raimondi F Meta-analysis of lung ultrasound scores for early prediction of bronchopulmonary dysplasia. Ann Am Thorac Soc. (2022) 19:659–67. 10.1513/AnnalsATS.202107-822OC 34788582

[19] Page MJ McKenzie JE Bossuyt PM Boutron I Hoffmann TC Mulrow CD The PRISMA 2020 statement: an updated guideline for reporting systematic reviews. Br Med J. (2021) 372:n71. 10.1136/bmj.n71 33782057 PMC8005924

[20] Whiting PF Rutjes AWS Westwood ME Mallett S Deeks JJ Reitsma JB QUADAS-2: a revised tool for the quality assessment of diagnostic accuracy studies. Ann Intern Med. (2011) 155:529–36. 10.7326/0003-4819-155-8-201110180-00009 22007046

[21] Cumpston M Li T Page MJ Chandler J Welch VA Higgins JP Updated guidance for trusted systematic reviews: a new edition of the cochrane handbook for systematic reviews of interventions. Cochrane Database Syst Rev. (2019) 10:ED000142. 10.1002/14651858.ED000142 31643080 PMC10284251

[22] Abdelmawla M Louis D Narvey M Elsayed Y. A lung ultrasound severity score predicts chronic lung disease in preterm infants. Am J Perinatol. (2019) 36:1357–61. 10.1055/s-0038-1676975 30609427

[23] Aldecoa-Bilbao V Velilla M Teresa-Palacio M Esponera CB Barbero AH Sin-Soler M Lung ultrasound in bronchopulmonary dysplasia: patterns and predictors in very preterm infants. Neonatology. (2021) 118:537–45. 10.1159/000517585 34515177

[24] Aliyev F Kayki G Annakkaya Kocyigit T İyigun İ Yigit S. Lung ultrasound scores within the first 3 days of life to predict respiratory outcomes. Pediatr Pulmonol. (2024) 59:662–8. 10.1002/ppul.26804 38131470

[25] Alonso-Ojembarrena A Serna-Guerediaga I Aldecoa-Bilbao V Gregorio-Hernández R Alonso-Quintela P Concheiro-Guisán A The predictive value of lung ultrasound scores in developing bronchopulmonary dysplasia: a prospective multicenter diagnostic accuracy study. Chest. (2021) 160:1006–16. 10.1016/j.chest.2021.02.066 33689782

[26] Alonso-Ojembarrena A Lubián-López SP. Lung ultrasound score as early predictor of bronchopulmonary dysplasia in very low birth weight infants. Pediatr Pulmonol. (2019) 54:1404–9. 10.1002/ppul.24410 31216121

[27] Gao S Xiao T Ju R Ma R Zhang X Xi Y The value of bedside lung ultrasound in predicting bronchopulmonary dysplasia in premature infants. Chin J Evid Based Med. (2020) 20:1385–9. 10.7507/1672-2531.202003020

[28] Ghanem M Zozaya C Ibrahim J Lee S Mohsen N Nasef N Correlation between early postnatal body weight changes and lung ultrasound scores as predictors of bronchopulmonary dysplasia in preterm infants: a secondary analysis of a prospective study. Eur J Pediatr. (2024) 183:2123–30. 10.1007/s00431-024-05464-z 38363393

[29] Hoshino Y Arai J Miura R Takeuchi S Yukitake Y Kajikawa D Lung ultrasound for predicting the respiratory outcome in patients with bronchopulmonary dysplasia. Am J Perinatol. (2022) 39:1229–35. 10.1055/s-0040-1721848 33374021

[30] Khandelwal S Datta V Anand R Devabathina NB. Role of lung ultrasound score in early prediction of bronchopulmonary dysplasia in preterm neonates. J Neonatal Perinatal Med. (2025) 18:52–60. 10.1177/19345798241296329 39973534

[31] Li Z Mu X Dang D Lv X Si S Guo Y Comparison of lung ultrasound scores with clinical models for predicting bronchopulmonary dysplasia. Eur J Pediatr. (2023) 182:1697–705. 10.1007/s00431-023-04847-y 36757494 PMC10167145

[32] Liu X Lv X Jin D Li H Wu H. Lung ultrasound predicts the development of bronchopulmonary dysplasia: a prospective observational diagnostic accuracy study. Eur J Pediatr. (2021) 180:2781–9. 10.1007/s00431-021-04021-2 33755776

[33] Loganathan PK Meau-Petit V Bhojnagarwala B Nair V Holmes J Occhipinti A Serial lung ultrasound in predicting the need for surfactant and respiratory course in preterm infants-multicentre observational study (SLURP). Eur J Pediatr. (2025) 184:356. 10.1007/s00431-025-06185-7 40407825 PMC12102118

[34] Loi B Vigo G Baraldi E Raimondi F Carnielli VP Mosca F Lung ultrasound to monitor extremely preterm infants and predict bronchopulmonary dysplasia. A multicenter longitudinal cohort study. Am J Respir Crit Care Med. (2021) 203:1398–409. 10.1164/rccm.202008-3131OC 33352083

[35] Martini S Gatelli IF Vitelli O Galletti S Camela F De Rienzo F Prediction of respiratory distress severity and bronchopulmonary dysplasia by lung ultrasounds and transthoracic electrical bioimpedance. Eur J Pediatr. (2023) 182:1039–47. 10.1007/s00431-022-04764-6 36562832

[36] Mohamed A Mohsen N Diambomba Y Lashin A Louis D Elsayed Y Lung ultrasound for prediction of bronchopulmonary dysplasia in extreme preterm neonates: a prospective diagnostic cohort study. J Pediatr. (2021) 238:187–192.e2. 10.1016/j.jpeds.2021.06.079 34237347

[37] Oulego-Erroz I Alonso-Quintela P Terroba-Seara S Jiménez-González A Rodríguez-Blanco S. Early assessment of lung aeration using an ultrasound score as a biomarker of developing bronchopulmonary dysplasia: a prospective observational study. J Perinatol. (2021) 41:62–8. 10.1038/s41372-020-0724-z 32665687 PMC7358564

[38] Teresa-Palacio M Avià X Balcells-Esponera C Herranz-Barbero A Alsina-Casanova M Carrasco C Accuracy of point-of-care nasopharyngeal interleukin 6 and lung ultrasound in predicting the development of bronchopulmonary dysplasia in preterm infants born before 30 weeks of gestation. PLoS One. (2025) 20:e0319739. 10.1371/journal.pone.0319739 40173116 PMC11964214

[39] Raimondi F Migliaro F Corsini I Meneghin F Dolce P Pierri L Lung ultrasound score progress in neonatal respiratory distress syndrome. Pediatrics. (2021) 147:e2020030528. 10.1542/peds.2020-030528 33688032

[40] Sun Y-H Du Y Shen J-R Ai D-Y Huang X-Y Diao S-H A modified lung ultrasound score to evaluate short-term clinical outcomes of bronchopulmonary dysplasia. BMC Pulm Med. (2022) 22(95):1–11. 10.1186/s12890-022-01885-4 35305612 PMC8933905

[41] Szymański P Puskarz-Gąsowska J Hożejowski R Stefańska M Błaż W Sadowska-Krawczenko I Prognostic relevance of the lung ultrasound score: a multioutcome study in infants with respiratory distress syndrome. Am J Perinatol. (2024) 41:e2862–9. 10.1055/s-0043-1775975 37848043 PMC11150063

[42] Zong H Huang Z Fu Y Chen X Yu Y Huang Y Lung ultrasound score as a tool to predict severity of bronchopulmonary dysplasia in neonates born ≤25 weeks of gestational age. J Perinatol. (2024) 44:273–9. 10.1038/s41372-023-01811-4 38087005

[43] Martini S Corsini I Corvaglia L Suryawanshi P Chan B Singh Y. A scoping review of echocardiographic and lung ultrasound biomarkers of bronchopulmonary dysplasia in preterm infants. Front Pediatr. (2023) 11:1067323. 10.3389/fped.2023.1067323 36846161 PMC9950276

[44] Demi L Wolfram F Klersy C De Silvestri A Ferretti VV Muller M New international guidelines and consensus on the use of lung ultrasound. J Ultrasound Med. (2023) 42:309–44. 10.1002/jum.16088 35993596 PMC10086956

[45] Wohlgemuth KJ Blue MNM Mota JA. Reliability and accuracy of ultrasound image analyses completed manually versus an automated tool. PeerJ. (2022) 10:e13609. 10.7717/peerj.13609 35729910 PMC9206842

[46] Angoulvant F Llor J Alberti C Kheniche A Zaccaria I Garel C Inter-observer variability in chest radiograph Reading for diagnosing acute lung injury in children. Pediatr Pulmonol. (2008) 43:987–91. 10.1002/ppul.20890 18702115

[47] Yadav S Lee B. Neonatal respiratory distress syndrome. StatPearls. Treasure Island, FL: StatPearls Publishing (2025). Available online at: http://www.ncbi.nlm.nih.gov/books/NBK560779/ (Accessed August 6, 2025) 32809614

[48] Thekkeveedu R K Guaman MC Shivanna B. Bronchopulmonary dysplasia: a review of pathogenesis and pathophysiology. Respir Med. (2017) 132:170–7. 10.1016/j.rmed.2017.10.014 29229093 PMC5729938

[49] Kim H-R Kim JY Yun BL Lee B Choi CW Kim BI. Interstitial pneumonia pattern on day 7 chest radiograph predicts bronchopulmonary dysplasia in preterm infants. BMC Pediatr. (2017) 17:125. 10.1186/s12887-017-0881-1 28506211 PMC5433188

