# Supplementary figures and images for: Diagnostic accuracy of lung ultrasound score for bronchopulmonary dysplasia in preterm neonates: a systematic review and meta-analysis
Source: Front Pediatr. 2025 Dec 17;13:1694150. doi: 10.3389/fped.2025.1694150 (PMC12753949; doi:10.3389/fped.2025.1694150)

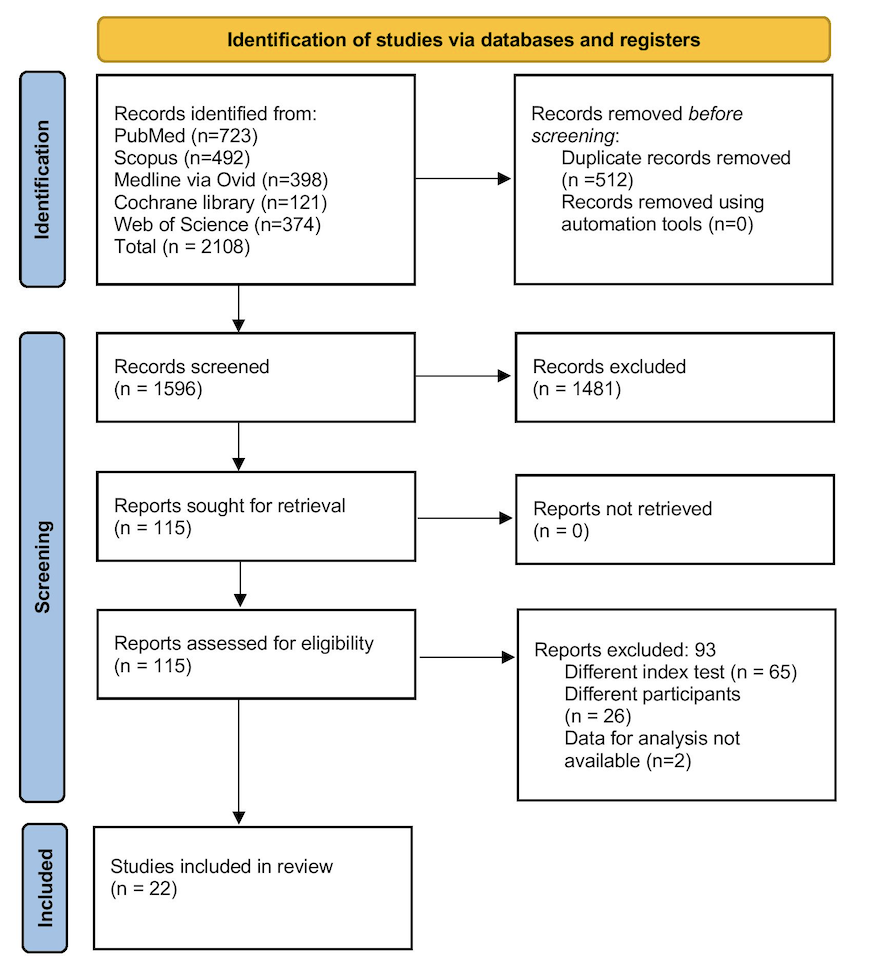

Supplement: Supplementary Figure S1 — PRISMA flowchart. [file Image1.tiff]

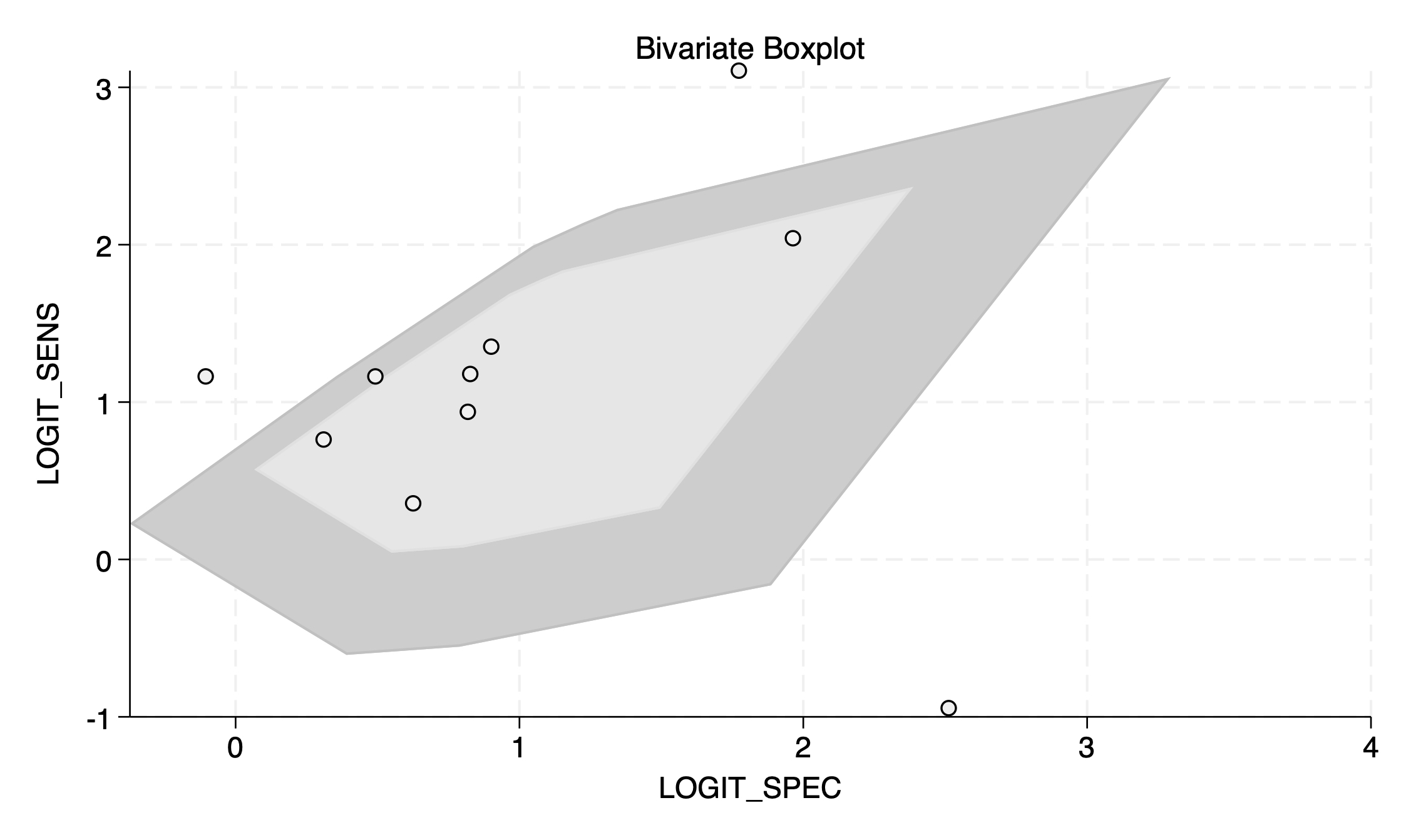

Supplement: Supplementary Figure S2 — Bivariate box plot for lung ultrasound scores for bronchopulmonary dysplasia at first three days of life. [file Image2.tiff]

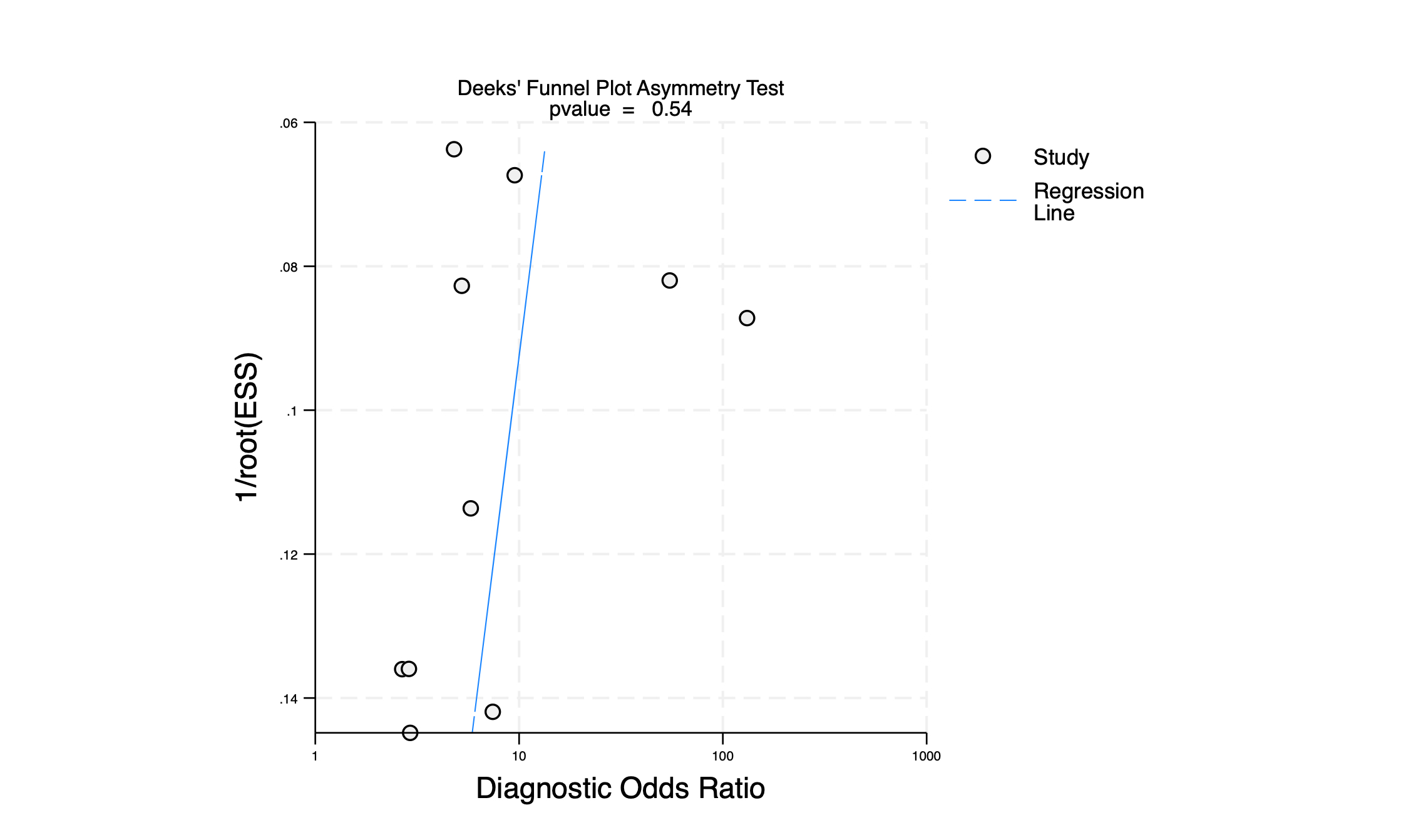

Supplement: Supplementary Figure S3 — Deek's funnel plot for lung ultrasound scores for bronchopulmonary dysplasia at first three days of life. [file Image3.tiff]

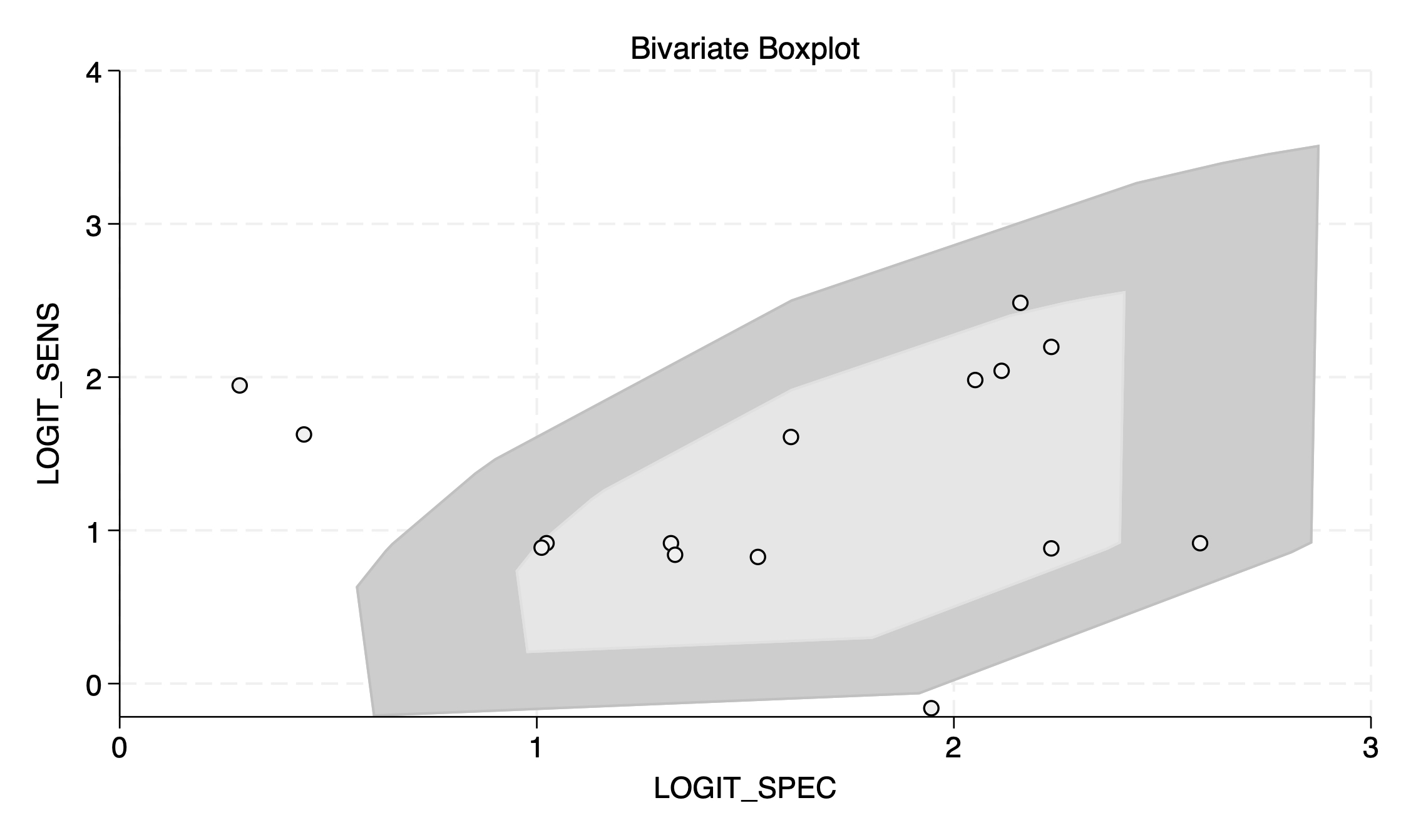

Supplement: Supplementary Figure S4 — Bivariate box plot for lung ultrasound scores for bronchopulmonary dysplasia at postnatal day 7. [file Image4.tiff]

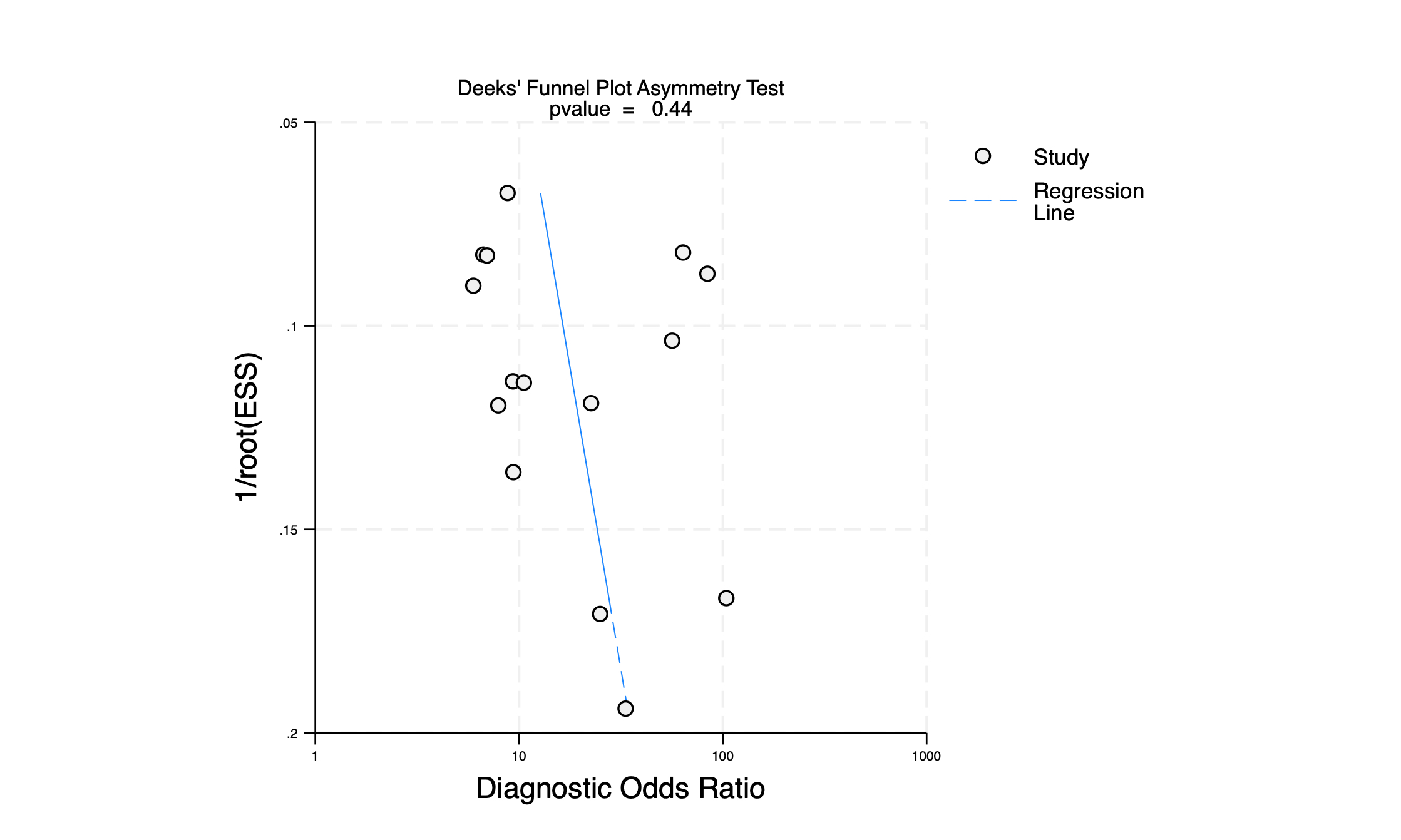

Supplement: Supplementary Figure S5 — Deek's funnel plot for lung ultrasound scores for bronchopulmonary dysplasia at postnatal day 7. [file Image5.tiff]

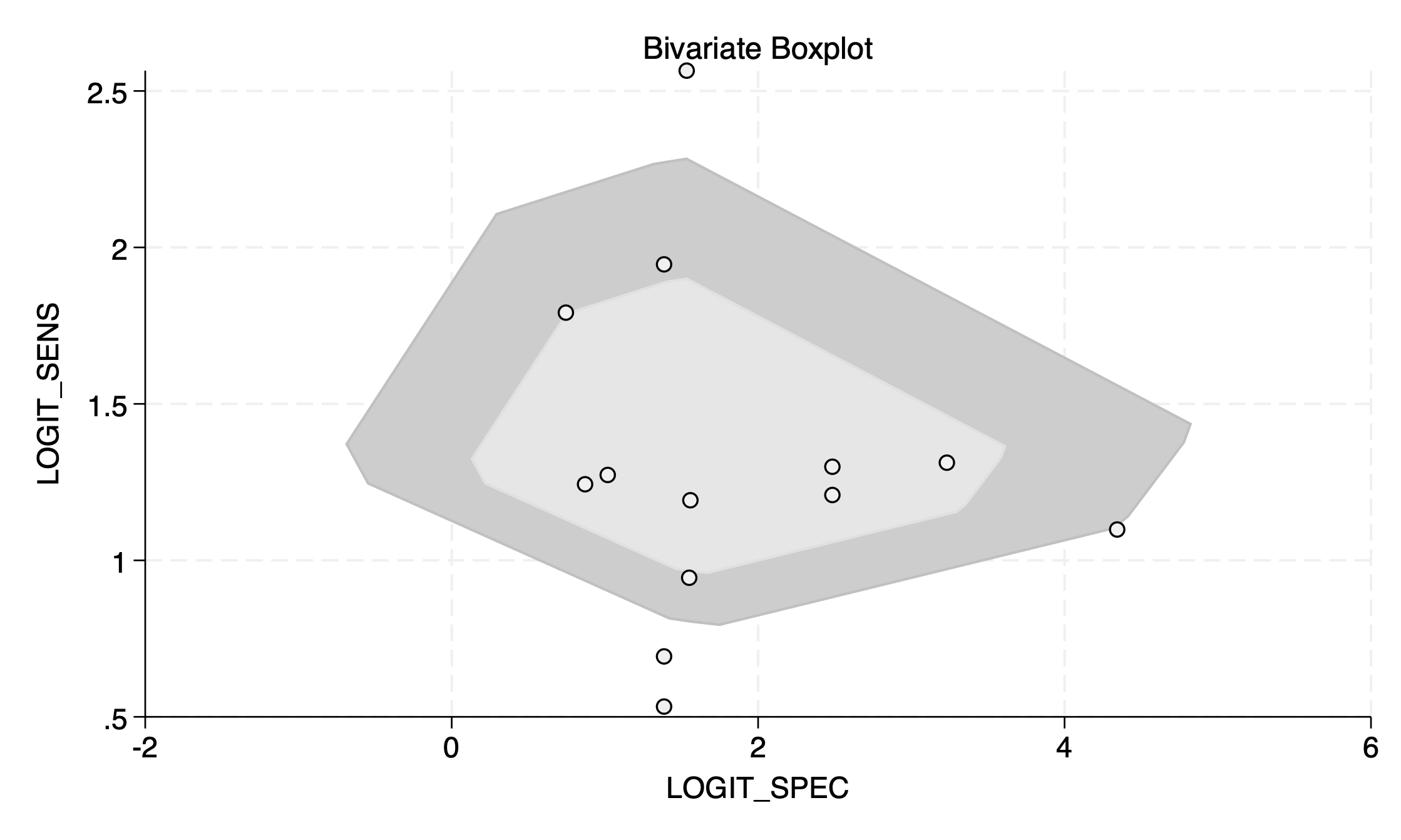

Supplement: Supplementary Figure S6 — Bivariate box plot for lung ultrasound scores for bronchopulmonary dysplasia at postnatal day 14. [file Image6.tiff]

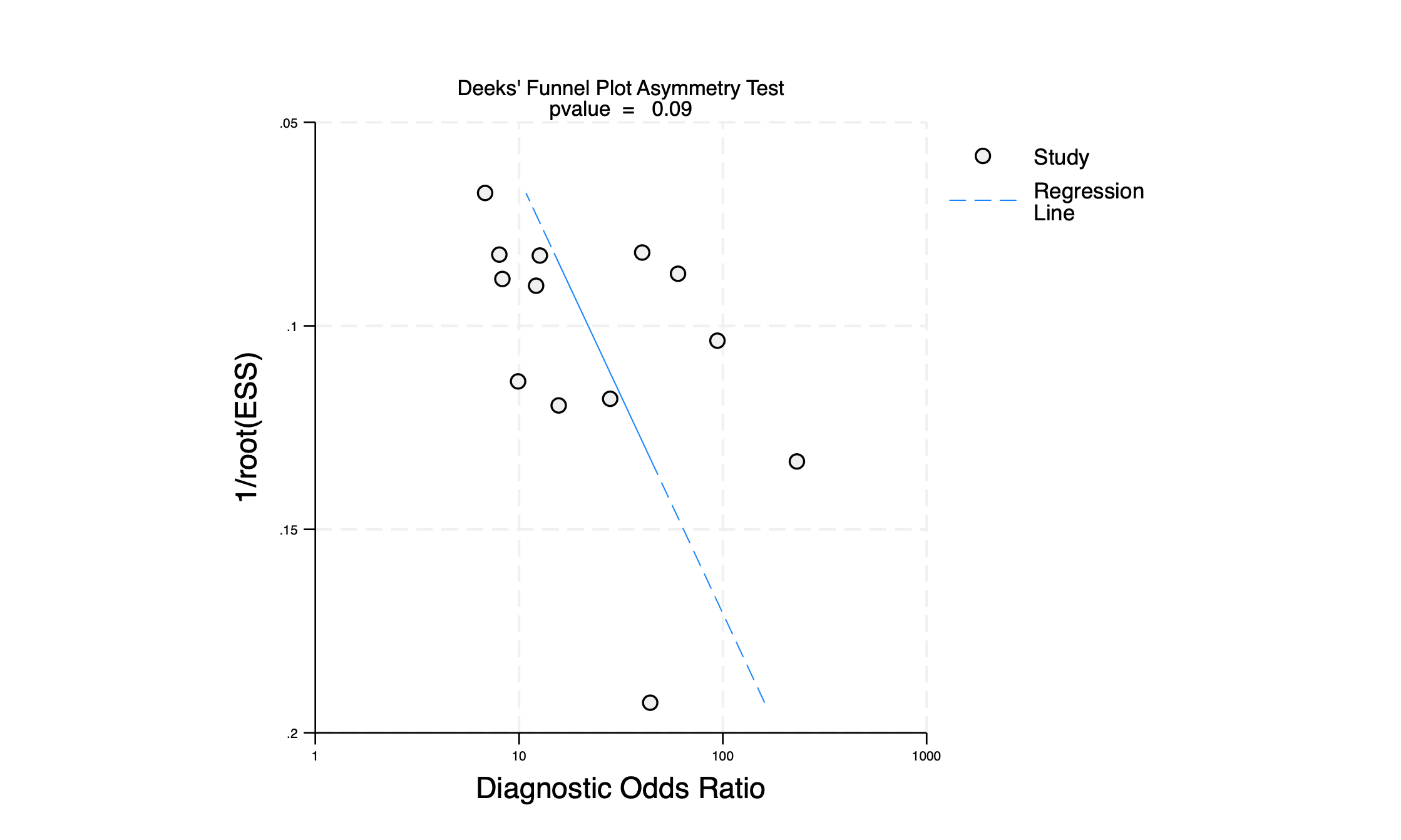

Supplement: Supplementary Figure S7 — Deek's funnel plot for lung ultrasound scores for bronchopulmonary dysplasia at postnatal day 14. [file Image7.tiff]

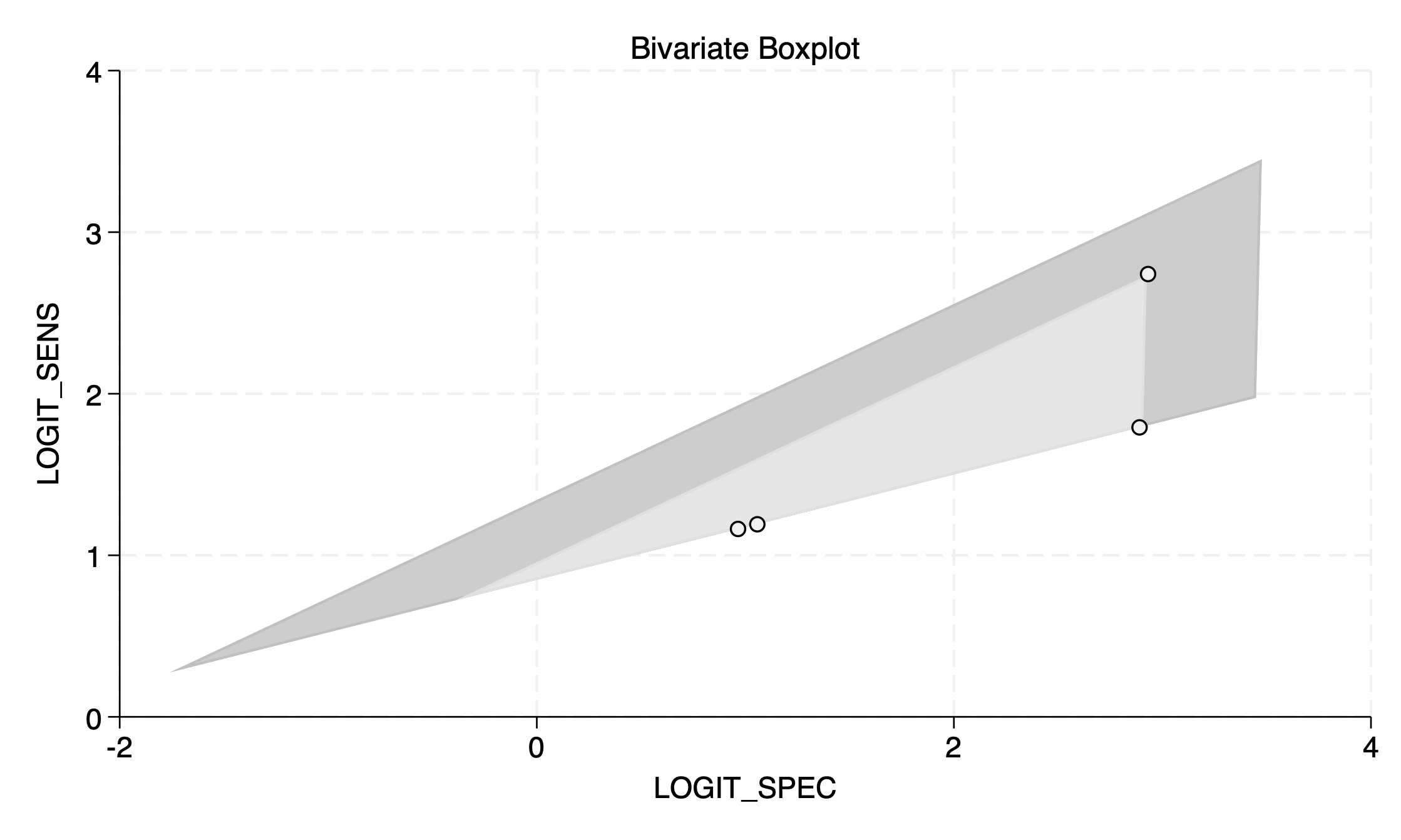

Supplement: Supplementary Figure S8 — Bivariate box plot for lung ultrasound scores for bronchopulmonary dysplasia at postnatal day 21. [file Image8.tiff]

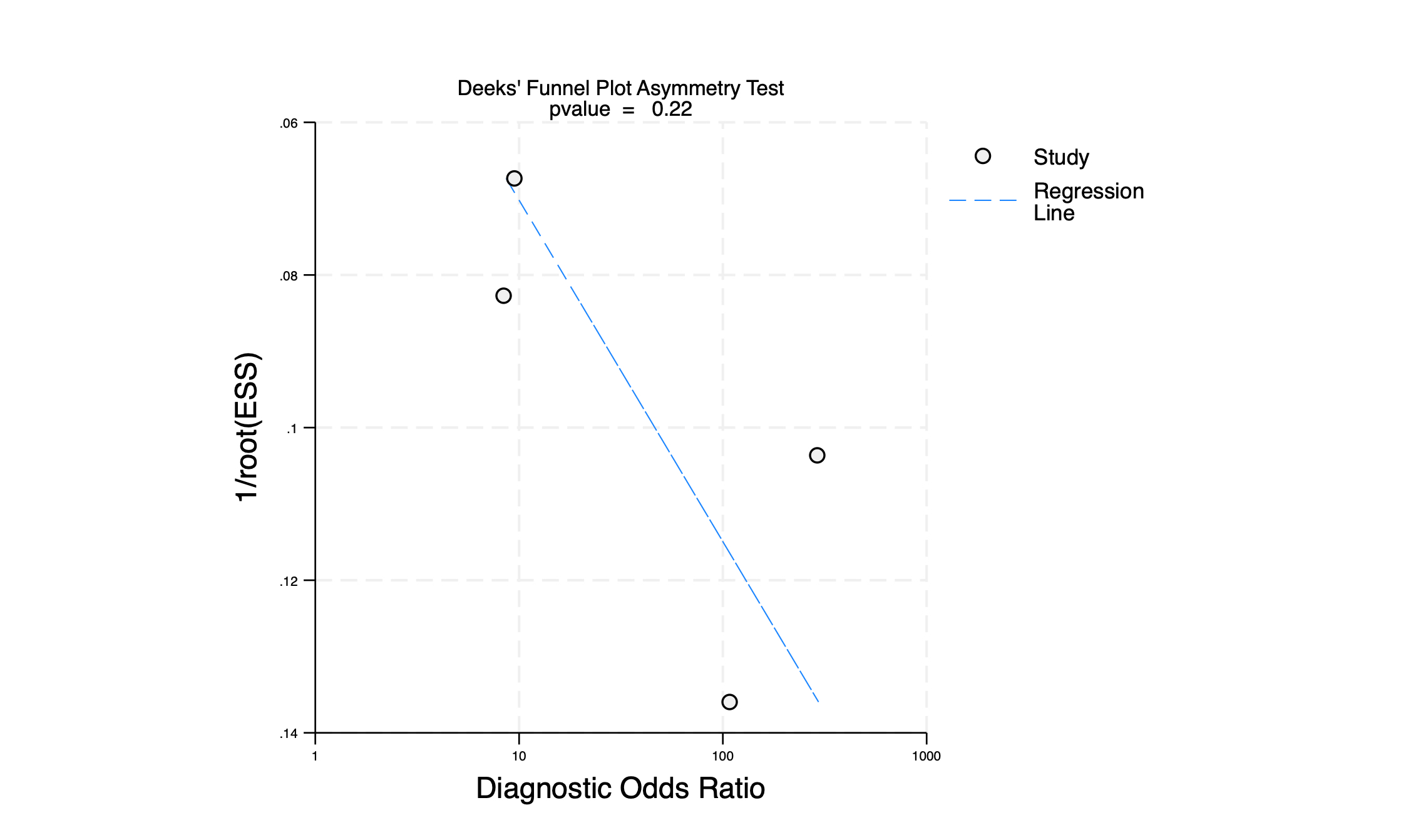

Supplement: Supplementary Figure S9 — Deek's funnel plot for lung ultrasound scores for bronchopulmonary dysplasia at postnatal day 21. [file Image9.tiff]
