# Supplementary material for: Diagnostic accuracy of lung ultrasound score for bronchopulmonary dysplasia in preterm neonates: a systematic review and meta-analysis
Source: Front Pediatr. 2025 Dec 17;13:1694150. doi: 10.3389/fped.2025.1694150 (PMC12753949; doi:10.3389/fped.2025.1694150)
Supplement: Supplementary file 10 [file Datasheet1.pdf]

Supplementary Appendix:

Search Strategy:

PubMed:

("Bronchopulmonary Dysplasia"[Mesh] OR bronchopulmonary dysplasia[tiab] OR BPD[tiab] OR "chronic lung disease of prematurity"[tiab] OR "oxygen dependency"[tiab] OR "respiratory distress syndrome survivors"[tiab])

AND ("Lung Ultrasonography"[Mesh] OR lung ultrasonography[tiab] OR lung ultrasound[tiab] OR LUS[tiab] OR "lung ultrasound score"[tiab] OR "LUS score"[tiab] OR "lung ultrasound severity score"[tiab] OR "lung ultrasound scoring"[tiab] OR "LUS scoring system"[tiab] OR semiquantitative[tiab] OR "pulmonary ultrasound"[tiab] OR "pulmonary ultrasonography"[tiab] OR "bedside lung ultrasound"[tiab] OR "neonatal lung ultrasound"[tiab] OR "neonatal LUS"[tiab])

AND ("Infant, Premature"[Mesh] OR premature[tiab] OR preterm[tiab] OR pre-term[tiab] OR neonate[tiab] OR newborn[tiab] OR neonatal[tiab] OR "very low birth weight"[tiab] OR VLBW[tiab] OR "extremely low birth weight"[tiab] OR ELBW[tiab])

AND ("Sensitivity and Specificity"[Mesh] OR sensitivity[tiab] OR specificity[tiab] OR accuracy[tiab] OR "diagnostic accuracy"[tiab] OR "predictive value"[tiab] OR ROC[tiab] OR "ROC curve"[tiab] OR AUC[tiab] OR "area under the curve"[tiab] OR "likelihood ratio"[tiab] OR "positive likelihood ratio"[tiab] OR "negative likelihood ratio"[tiab] OR "diagnostic odds ratio"[tiab] OR "post-test probability"[tiab] OR "Fagan nomogram"[tiab] OR "receiver operating characteristic"[tiab])

Medline via Ovid:

(exp Bronchopulmonary Dysplasia/ OR bronchopulmonary dysplasia.mp. OR BPD.mp. OR CLD.mp. OR "chronic lung disease of prematurity".mp. OR oxygen dependency.mp. OR "respiratory distress syndrome survivors".mp.) AND (exp Lung Ultrasonography/ OR lung ultrasonography.mp. OR lung ultrasound.mp. OR "lung US".mp. OR LUS.mp. OR "lung sonography".mp. OR "thoracic ultrasound".mp. OR "thoracic ultrasonography".mp. OR "pulmonary ultrasound".mp. OR pulmonary ultrasonography.mp. OR "chest ultrasound".mp. OR semiquantitative.mp. OR "lung ultrasound score".mp. OR "LUS score".mp.) AND (exp Infant, Premature/ OR preterm.mp. OR premature.mp. OR "pre-term".mp. OR "very low birth weight".mp. OR VLBW.mp. OR "extremely low birth weight".mp. OR ELBW.mp. OR neonate.mp. OR newborn.mp. OR neonatal.mp.) AND (exp Sensitivity and Specificity/ OR sensitivity.mp. OR specificity.mp. OR accuracy.mp. OR "diagnostic accuracy".mp. OR "predictive value".mp. OR ROC.mp. OR AUC.mp. OR "area under the curve".mp. OR "likelihood ratio".mp. OR "positive likelihood ratio".mp. OR "negative likelihood ratio".mp. OR "diagnostic odds ratio".mp. OR DOR.mp. OR "post-test probability".mp. OR "Fagan nomogram".mp. OR "LR scattergram".mp.)

Scopus:

TITLE-ABS-KEY(("Bronchopulmonary Dysplasia" OR bronchopulmonary dysplasia OR BPD OR "chronic lung disease of prematurity" OR "oxygen dependency" OR "respiratory distress syndrome survivors") AND ("Lung Ultrasonography" OR lung ultrasonography OR lung ultrasound OR LUS OR "lung ultrasound score" OR "LUS score" OR "lung ultrasound severity score" OR "lung ultrasound scoring" OR "LUS scoring system" OR semiquantitative OR "pulmonary ultrasound" OR "pulmonary ultrasonography" OR "bedside lung ultrasound" OR "neonatal lung ultrasound") AND (preterm OR premature OR "very low birth weight"

OR VLBW OR "extremely low birth weight" OR ELBW OR neonate OR newborn OR neonatal) AND (sensitivity OR specificity OR accuracy OR "diagnostic accuracy" OR "predictive value" OR ROC OR AUC OR "area under the curve" OR "likelihood ratio" OR "positive likelihood ratio" OR "negative likelihood ratio" OR "diagnostic odds ratio" OR "post-test probability" OR "Fagan nomogram" OR "receiver operating characteristic"))

Web of Science:

TS=("Bronchopulmonary Dysplasia" OR bronchopulmonary dysplasia OR BPD OR "chronic lung disease of prematurity" OR "oxygen dependency" OR "respiratory distress syndrome survivors") AND TS=("Lung Ultrasonography" OR lung ultrasonography OR lung ultrasound OR LUS OR "lung ultrasound score" OR "LUS score" OR "lung ultrasound severity score" OR "lung ultrasound scoring" OR "LUS scoring system" OR semiquantitative OR "pulmonary ultrasound" OR "pulmonary ultrasonography" OR "bedside lung ultrasound" OR "neonatal lung ultrasound") AND TS=(preterm OR premature OR "very low birth weight" OR VLBW OR "extremely low birth weight" OR ELBW OR neonate OR newborn OR neonatal) AND TS=(sensitivity OR specificity OR accuracy OR "diagnostic accuracy" OR "predictive value" OR ROC OR AUC OR "area under the curve" OR "likelihood ratio" OR "positive likelihood ratio" OR "negative likelihood ratio" OR "diagnostic odds ratio" OR "post-test probability" OR "Fagan nomogram" OR "receiver operating characteristic")

Cochrane library:

("Bronchopulmonary Dysplasia":ti,ab,kw OR bronchopulmonary dysplasia:ti,ab,kw OR BPD:ti,ab,kw OR "chronic lung disease of prematurity":ti,ab,kw OR "oxygen dependency":ti,ab,kw OR "respiratory distress syndrome survivors":ti,ab,kw) AND ("Lung Ultrasonography":ti,ab,kw OR lung ultrasound:ti,ab,kw OR LUS:ti,ab,kw OR "lung

ultrasonography":ti,ab,kw OR "pulmonary ultrasound":ti,ab,kw OR "thoracic ultrasound":ti,ab,kw OR semiquantitative:ti,ab,kw OR "lung ultrasound score":ti,ab,kw OR "LUS score":ti,ab,kw) AND (preterm:ti,ab,kw OR premature:ti,ab,kw OR neonate:ti,ab,kw OR newborn:ti,ab,kw OR neonatal:ti,ab,kw OR VLBW:ti,ab,kw OR ELBW:ti,ab,kw) AND (sensitivity:ti,ab,kw OR specificity:ti,ab,kw OR accuracy:ti,ab,kw OR "diagnostic accuracy":ti,ab,kw OR "predictive value":ti,ab,kw OR ROC:ti,ab,kw OR AUC:ti,ab,kw OR "likelihood ratio":ti,ab,kw OR "Fagan nomogram":ti,ab,kw OR "diagnostic odds ratio":ti,ab,kw OR "post-test probability":ti,ab,kw) AND lang:english
